# Assessment of Elemental Deficiency of Crossbred Dairy Cows and Mineral Composition in Natural Feed and Nutritional Supplements in the Northern and Northwestern Provinces in Sri Lanka

**DOI:** 10.1007/s12011-024-04299-x

**Published:** 2024-07-12

**Authors:** Joseph W. Clarkson, Neil I. Ward, Joaquín M. Prada, David Tisdall, Mónica Felipe-Sotelo, J. K. Vidanarachchi, Mike Christian, Mark Andrew Chambers

**Affiliations:** 1 School of Chemistry and Chemical Engineering, University of Surrey, Stag Hill Campus, Guildford, Surrey GU2 7XH UK; 2 Faculty of Health and Medical Sciences, School of Veterinary Medicine, University of Surrey, Manor Park Campus, Daphne Jackson Road, Guildford, GU2 7AL UK; 3 Department of Animal Science, University of Peradeniya, Peradeniya, 20400 Sri Lanka; 4 Vetbiz Consultancy, Wigton, CA7 0LW UK

**Keywords:** Trace elements, Bovine milk, Bovine blood, Forage, Feed supplements

## Abstract

This study assessed the elemental status of cross-bred dairy cows in small holder farms in Sri Lanka, with the aim to establish the elemental baseline and identify possible deficiencies. For this purpose, 458 milk, hair, serum and whole blood samples were collected from 120 cows in four regions of Northern and Northwestern Sri Lanka, (namely Vavaniya, Mannar, Jaffna and Kurunegala). Farmers also provided a total of 257 samples of feed, which included local fodder as well as 79 supplement materials. The concentrations of As, Ca, Cd, Co, Cr, Cu, Fe, I, K, Mg, Mn, Mo, Na, Ni, Pb, Se, V and Zn were determined by inductively coupled plasma mass spectrometry (ICP-MS). Evaluation of the data revealed that all cows in this study could be considered deficient in I and Co (18.6–78.5 µg L^−1^ I and 0.06–0.65 µg L^−1^ Co, in blood serum) when compared with deficiency upper boundary levels of 0.70 µg L^−1^ Co and 50 µg L^−1^ I. Poor correlations were found between the composition of milk or blood with hair, which suggests that hair is not a good indicator of mineral status. Most local fodders meet dietary requirements, with Sarana grass offering the greatest nutritional profile. Principal component analysis (PCA) was used to assess differences in the elemental composition of the diverse types of feed, as well as regional variability, revealing clear differences between forage, concentrates and nutritional supplements, with the latter showing higher concentrations of non-essential or even toxic elements, such as Cd and Pb.

**Supplementary Information:**

The online version contains supplementary material available at 10.1007/s12011-024-04299-x.

## Introduction

During the Sri Lankan civil war, which ended in 2009, Northern Sri Lanka lost approximately 50–60% of its dairy cattle. Moreover, climate changes in recent decades have been affecting the rainfall in the region, making rice farming on paddy fields less reliable and sustainable. Sri Lanka currently imports 60% of the milk it consumes as dried milk powder [1]. As a result, government policies have been promoting a move towards dairy farming in rural areas. To promote self-sufficiency as a country, various governmental, non-governmental and private sector organisations in Sri Lanka have offered financial support to encourage dairy farming. The Department of Animal Production and Health (DAPH) subsidises insemination services and vaccinations for dairy cattle. However, one factor limiting the growth of the sector is poor fertility, which is a major driver of dairy production.

There is some anecdotal evidence in the studied area to suggest that mineral or elemental deficiencies are present because, when given supplementation, fertility has improved. Nevertheless, it is possible that mineral supplementation has coincided with cows returning to energy balance post-calving or other confounding factors contributing to improved fertility. Although trace element disorders are a well-known cause of production problems for the dairy industry, they may be carrying more than their fair share of blame for poor cattle performance [2]. Indeed, they are the least probable explanation for any production or fertility issue. Nevertheless, they are important, especially since the requirements for dairy cattle in the tropics in smallholder farms are extrapolated from the very different intensive dairy farms in temperate regions. There is little research to establish nutritional baselines or the relevance of trace elements in fertility disorders in locations in the tropics, and there have been only a few publications so far on the elemental status of dairy cows, focusing only on Se [3] and bovine milk [4, 5] in Sri Lanka.

The trace element requirements and levels for cattle are variable. There is not a definitive range that can be used. Trace element requirements vary according to age [6], sex [7], growth stage [8] and breed and genotype [9]. It is expected that different breeds would have slightly different dietary requirements in the same setting. However, data for indigenous or other breeds in tropical and sub-tropical climates are very limited [10, 11]. Furthermore, rumen fermentation varies according to the presentation of the diet and the water availability. Hence, free-grazing cows selecting plants will differ from zero-grazing cows (cut and fed) given the same diet; also, diets presented as whole in the trough will be fermented in the rumen differently to mashes or diets that are well chopped and mixed.

The aim of the current study in Sri Lanka was to establish the baseline elemental status of smallholder dairy cows and their main feeds and supplements to identify any obvious deficiencies; hence, that evidence-based veterinary advice can be given. As it is difficult to obtain meaningful data from small farms because of the variable nature of the observations, data were collated across a range of farmers in the villages of Jaffna, Mannar, Kurunegala and Vavuniya in the Northern and the Northwestern Provinces of Sri Lanka, in order to obtain more representative and meaningful information.

## Experimental Methodology

### Sampling

**Fig. 1:**
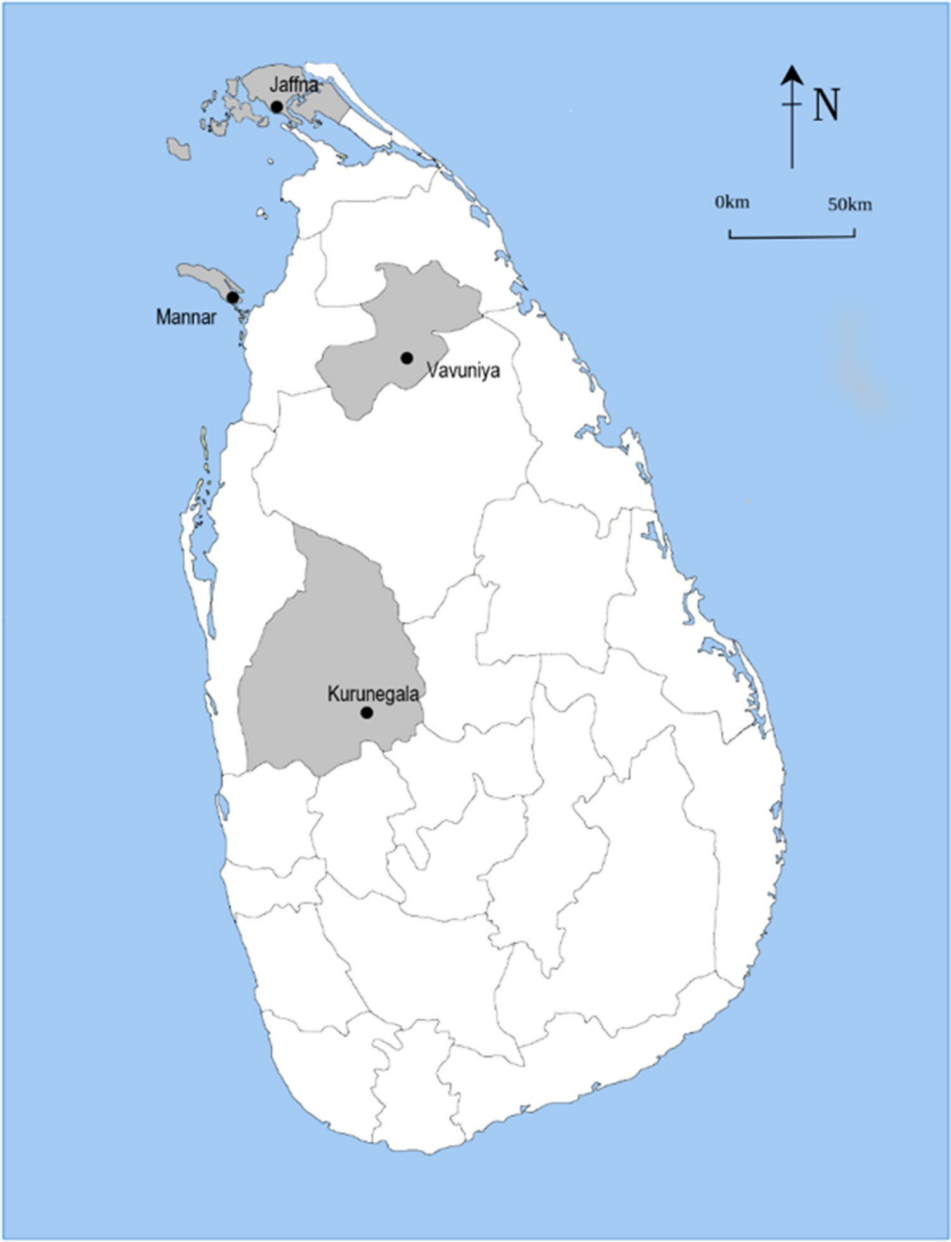
Map of Sri Lanka showing the location of the four geographical areas surveyed

This study was given ethical approval by the University of Surrey NASPA committee (ref: NERA-1819–078) and in Sri Lanka from the Provincial Council of DAPH (Department of Animal Production and Health). Samples were collected from small-scale dairy farms located in four regions of the Northern and Northwestern Provinces of Sri Lanka (Vavuniya, Mannar, Jaffna and Kurunegala, see locations on the map in Fig. 1). Jaffna, Mannar and Vavuniya are in the North of the country, within the ‘dry zone’ (ecoregion of the island of Sri Lanka characterised by tropical dry evergreen, broadleaf forests). The province of Kurunegala is in Northwestern Sri Lanka, within the ‘intermediate zone’, situated between the ‘dry zone’ in the North of the island and the ‘wet zone’ of the Southwest. Due to its large population, Kurunegala is viewed as a key site for veterinary provisions across Northwestern Sri Lanka. These four areas were selected to represent diverse small-scale dairy farming practices across different agro-climatological regions in Sri Lanka. In these regions, dairy farming amongst small-scale dairy farmers is practiced using a diverse range of natural feeds and nutritional supplements as well as management strategies, types of cattle breeds and socio-cultural aspects.

#### Blood, Serum, Hair and Milk Samples

In collaboration with local veterinarians from the DAPH, milk, hair, serum and whole blood samples were collected from 30 cows in each of the four locations (Fig. 1), totalling 458 samples from 120 dairy cows. The cows from multiple small-scale dairy farms were walked using a halter to a common point in the community where samples were taken. Blood samples were collected in screw-top containers and preloaded with EDTA to avoid coagulation (Na_2_EDTA, approx. 15 mg per mL of sample). The serum samples were prepared in situ, after letting the blood coagulate at room temperature for ca. 15–30 min, after which the supernatant was collected. Blood, serum and milk samples were kept refrigerated at < 4 °C during transport and until analysis. Hair samples were kept in 0.1-mL propyl alcohol for sterilisation prior to analysis.

The breed, colour and body condition scores (BCS) and fertility parameters were also recorded. These included age, date of first calving, number of calvings, pregnancy status and number of services. The BCS was assessed in accordance with the Penn-State method [12] (Table S1 of Supplementary Information, SI). The main breeds are native crosses with Sahiwal, Jersey or Friesian. For comparative purposes, samples were collected using the same procedures from six cows in Wigton (UK), selected as a control area.

#### Pasture and Fodder Samples

Forage and feed samples were provided by the farmers. In total, 257 samples were received for trace element determination; 79 of these samples were classified as feed or supplement materials, which are given as an additive to the grazing diet of dairy cattle. The remaining samples were classified as natural fodder, representative of the local plant species that make up the bulk of the diet of all dairy cattle in the region. These included forages (bought and harvested): paddy straw, Napier grass-CO3/CO4 (*Pennisetum perpureum* X *Pennisetum americarnum*), *Azolla* (mosquito fern, duckweed fern, fairy moss, water fern), Guinea grass (*Panicum maximum*) and the legume *Gliricidia* (*Gliricidia sepium*). More detailed information on the fodder and concentrates collected is available in Table S2 of the Supplementary Information (SI). Information recorded as provided by the farmers included the date of the last mineral supplement and minerals used and all quantities fed of forage and concentrates.

Forage, concentrates and nutritional supplement samples were received by the University of Surrey according to importation and licencing laws of the UK. Each individual sample was preserved within a labelled zip-locked plastic bag and stored at ambient temperature, between 18 and 30 °C.

### Sample Preparation

Upon arrival to the laboratories in the UK, the samples of whole blood, serum and milk were diluted in a solution of 0.6% v/v NH_4_OH (Honeywell Fluka™), 0.8% v/v H_2_O_2_ (Perdogen™ 30% w/w H_2_O_2_ puriss.), 0.01% v/v Triton X-100 (Alfa Aesar™) and 0.1% w/v EDTA (Na_2_EDTA·2H_2_O, certified AR for Analysis, Fisher Chemical™), ready for analysis by inductively coupled plasma mass spectrometry (ICP-MS) [13]. This procedure was used for all analytes in this study, with the exception of iodine, for which samples were diluted with 0.5% TMAH (tetramethylammonium hydroxide, 25% in water, Acros Organics™) [14]. In all cases, samples were kept refrigerated (4 °C) until instrumental analysis.

Before analysis, the samples of hair and fodder were washed to ensure the removal of exogenous elements covering the surface of the samples (i.e. any traces of soil or dust). The samples of fodder were washed repeatedly with deionised water and let to dry at room temperature. In the case of hair, the samples were washed according to the standardised procedure recommended by the International Atomic Energy Agency (IAEA). Hair samples were washed manually for 5 min with acetone and after decanting the washing liquid, three further washing steps were performed using deionised water, followed by a final washing step using acetone [15]. The cleaned hair samples were allowed to dry at room temperature before further treatment. The analysis of the elemental composition of hair samples and livestock feeds by ICP-MS required the extraction of the analytes into a solution suitable for introduction into the ICP. For this purpose, two different extraction methods were applied; the first was based on ashing of the materials followed by acid treatment in an open vessel [16], and the second was alkaline microwave–assisted digestion, specifically applied for iodine determination [17]. For the acid-based extraction; each solid sample was dried in an oven for 24 h at 60 °C. Following homogenisation of each dried sample (hair or feed) with pestle and mortar, 0.2000–0.2500 g dry weight (d.w.) was placed into pre-weighed acid-washed crucibles. The crucibles were then transferred into a muffle furnace (Carbolite, UK) and heated at approximately 550 °C for 12 h, following a gradual increase from room temperature. Upon the completion of the ashing procedure, the samples were removed from the furnace and allowed to cool to room temperature. Within a fume hood, approximately 1 mL of trace metal analysis grade concentrated HNO_3_ (Fisher Scientific, Loughborough, UK) was carefully added to each crucible, which was then transferred to a pre-weighed 25-mL centrifuge tube. The crucible was then washed with double-distilled water (DDW) multiple times and decanted into the centrifuge tube to ensure the transfer of all the digested solutions. The total volume of the sample was made up to 25 mL volume with DDW before the weight was recorded accurately in an analytical balance (± 0.0001). Finally, 5 mL of the solution was filtered through a 0.45-µm syringe-top filter (Millex, MilliporeTM, USA) before analysis by ICP-MS.

In the case of the extraction of iodine from hair, samples were treated in a microwave oven (MARS Xpress, CEM Microwave Technology Ltd, Buckingham, UK), based on the procedure reported by Jerše and collaborators [17]; 5 mL of 5% TMAH were added to the hair and heated up to 70 °C over 10 min, after which the temperature was kept at 70 °C for an hour. Once cooled to < 50 °C, the samples were transferred to 15-mL Nalgene tubes (Thermo Scientific™). The microwave reactors were rinsed with 5 mL of DDW, and the washing was added to the previous fraction into 15-mL Nalgene tubes. Finally, the solutions were centrifuged at 4500 × g for 20 min, and 5–8 mL aliquots of the supernatant were withdrawn for instrumental analysis.

For all preparation procedures described here, method blanks were performed in the same manner as per digestion of the samples and were measured as indicated in the “Instrumental Analysis and Validation” section to evaluate the limits of detection of the analytical methods.

### Instrumental Analysis and Validation

Element concentrations were analysed using an Agilent 7800 × Series ICP-MS with MassHunter Workstation software (Agilent Technologies, Stockport, UK). The range of elements assessed in this study included As, Ca, Cd, Co, Cr, Cu, Fe, I, K, Mg, Mn, Mo, Na, Ni, Pb, Se, V and Zn. Operation conditions for the instrumentation are given in Table S3. Optimisation and tuning of the ICP-MS were performed daily, and the instrument was operated in collision-cell mode (with He gas) for the elimination of polyatomic interferences. A range of multi-element standards from 1 to 750 µg/L was prepared in 1% HNO_3_ (v/v) from a 1000 mg/L standard stock solution (Aristar, Fisher Scientific, UK) for each of the analytes, except I for which separate sets of standards were prepared in 0.5% TMAH. A 100-µg/L mixture of internal standards (Sc, Ge, Rh and Bi; Agilent Technologies, UK) was injected into the plasma simultaneously with the samples and calibration standards to compensate for any drift in signal intensity during the ICP-MS analyses.

The accuracy of the analysis and instrumental performance was evaluated on a daily basis using two certified reference materials (CRMs), SRM 1640a and SRM 1643f (trace elements in natural water) from the National Institute of Standards and Technology (NIST). The results of the accuracy tests as well as the values of the instrument and method limits of detection (LOD) can be found in Table S4.

### Principal Components Analysis (PCA)

In order to assess whether there were significant differences in the elemental composition of the forage from the four geographical areas sampled in this study, principal components analysis (PCA) was performed on the results of the complete set of data, as well as to evaluate the differences in composition between the three types of feed, namely forage, concentrates and nutritional supplements. Additionally, PCA can provide insight into the relationships within the groups of elements determined in the animal feed. The commercial software package Matlab (MathWorks UK, Cambridge, UK) was used throughout to develop the PCA models. In the case of the experimental results that were below the limit of detection (LOD), values were substituted by half the LOD, as recommended by Farnham et al. [18]. For PCA, the data for each element were auto-scaled by subtracting the average for each elemental concentration and then dividing by the standard deviation.

## Results and Discussion

The technique used in this study, inductively coupled plasma mass spectrometry (ICP-MS), is used routinely to identify the elemental levels in many sample plants, soil contamination, etc., because of its great versatility, broad range of elements that can be determined simultaneously, wide linear dynamic range and excellent sensitivity. However, the standard techniques for the blood sampling of cattle focus on the important mineral-containing enzymes or proteins. Normal practice would be for Se glutathione peroxidase; for Co, cobalamin/vitamin B12 and for I, T4 (thyroxine) levels. These were not available in this project; hence, discussion will be based on the total elemental content in all matrices analysed. Due to the large quantity of information, the discussion of results in the “Elemental Deficiency of Crossbred Cows: Analysis of Whole Blood, Serum, Hair and Milk” and “Mineral Composition of Cow Feed: Forage, Concentrates and Nutritional Supplements” sections is focused on Co, Cu, Se and I, due to the role of these specific elements in the health and productivity of cattle and Mo, because of its antagonistic effect on Cu absorption [19, 20]. The complete set of data was employed for the chemometric modelling by PCA in the “Principal Components Analysis” section. A summary of the results for all analytes can be found in Tables S5 to S11.

### Elemental Deficiency of Crossbred Cows: Analysis of Whole Blood, Serum, Hair and Milk

**Fig. 2:**
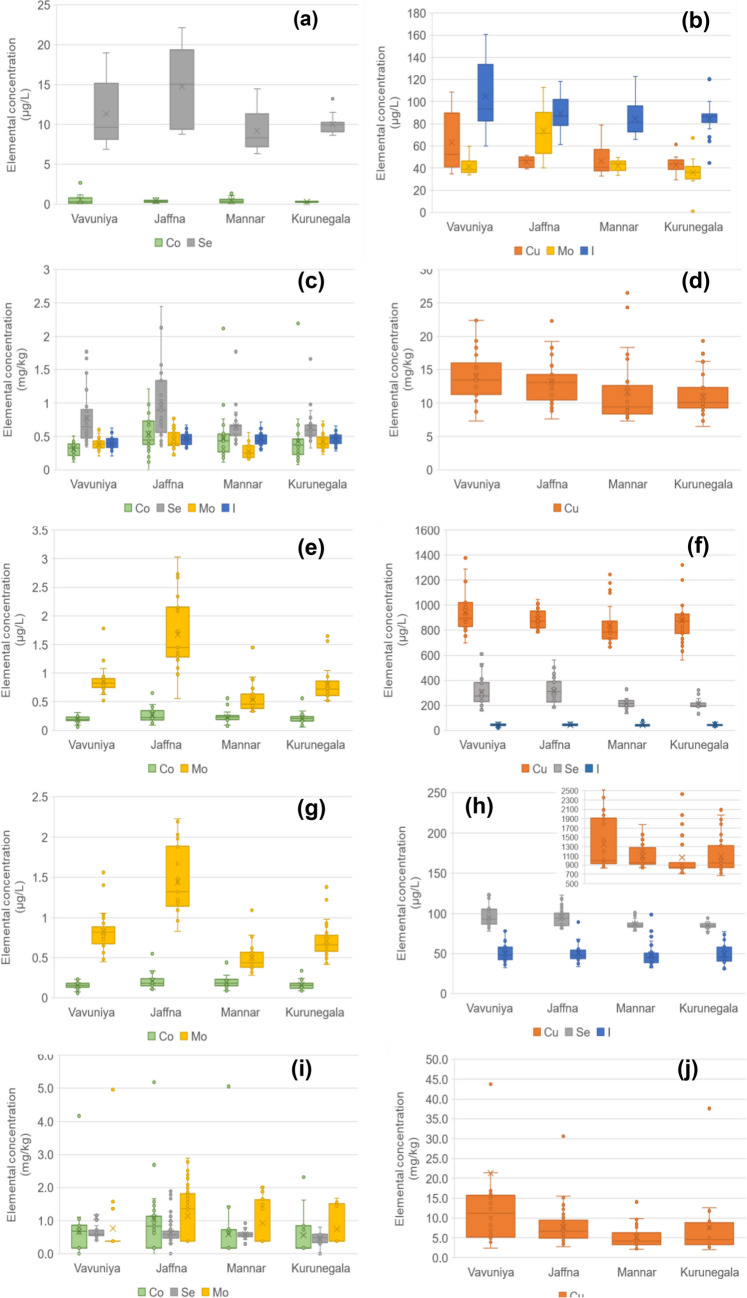
Box-plot representation of the levels of Co, Cu, Se, Mo and I in the four biomarkers **a**, **b** milk, **c**, **d** hair, **e**, **f** blood serum and **g**, **h** whole blood and **i**, **j** forage feed in the regions of Vavuniya, Jaffna, Mannar and Kurunegala. Note the logarithmic scale on the concentration axis to allow for simultaneous visualisation of all elements

In this project, the following terms are used: (i) deficient, levels at which clinical or pathological signs of element deficiency should be apparent in some individuals; (ii) marginal, levels at which subclinical effects may prevail, such as reduced immune response, or reduced growth rate; (iii) adequate, levels sufficient for optimum functioning of all body mechanisms with a small margin of reserve to counteract commonly encountered antagonistic conditions; (iv) high, levels elevated well above normal but not necessarily toxic, and (v) toxic, levels at which subclinical, clinical or pathological signs of toxicity would be expected to occur [2, 8, 21, 22]. The boundary values are summarised in Table 1. According to these criteria, all the cows evaluated in this study could be considered deficient in I (values ranging from 18.6 to 78.5 µg/L I in blood serum, Table S6) and Co (0.06–0.65 µg/L Co in blood serum, Table S6). Low serum Co can be an indicator of low vitamin B12; however, it should be noted that in this study, only total Co was determined. Under the assumption that Co is only present as vitamin B12, the concentration of Co, presented here in micrograms per liter, can be easily converted to vitamin B12 by multiplying by 1355/58.9 [2]. While all the Cu levels in blood serum were adequate (563–1376 µg/L Cu), all the Se levels ranged from adequate to high (132–609 µg/L Se) especially in Vavuniya (304 ± 110 µg/L Se, *n* = 30) and Jaffna (312 ± 102 µg/L Se, *n* = 30). However, low levels of Mo were found in all biological samples. Data for Co, Cu, Se, Mo and I are represented as box plots in Fig. 2. A summary of the results for all elements in the biological samples can be found in Tables S5–S8. From the data in Fig. 2, it is not possible to establish any notable difference in the levels of Co, Cu, Se, Mo and I across the four geographical regions under investigation, with an overlap of all the concentration ranges. As can be seen in Fig. 2e and f for blood serum and Fig. 2g and h for whole blood, the variation of these elements is much narrower than for milk (Fig. 2a and b) and hair (Fig. 2c and d), which seems to suggest homeostatic control of these elements.

**Table 1:** Boundary values (in part per billion, ppb) for trace element in blood serum of dairy cows and number of cows within each category (total number 120)

		Elemental concentration (ppb)														
		Co			Cu			Mo			Se			I		
		LL	UP	NoC	LL	UP	NoC	LL	UP	NoC	LL	UP	NoC	LL	UP	NoC
Boundary levels	Deficient	LOD	0.70	120	LOD	550	0	LOD	10.00	120	2	25	0	10	50	97
	Deficient to marginal	-	-	-	-	-	-	-	-	-	25	30	0	50	80	23
	Marginal	0.70	0.90	0	550	599	1	-	-	0	30	80	0	80	100	0
	Adequate	0.90	15	0	600	1500	119	10	100	0	80	300	90	100	400	0
	High	15	3000	0	>1500	-	0	>100	-	0	300	3500	30	700	300	0
	Toxic	3000	-	0	-	-	0	-	-	0	>3500	-	0	>3000	25000	0

Boundary limits taken from references [2, 8, 21, 22]

*LOD* limit of detection, *LL* lower limit, *UL* upper limit, *NoC* number of cows

The results of Pearson correlations [23] between the concentrations of Co, Cu, Se, Mo and I in the four biological matrices can be found in Table 2. At 99% confidence level, all correlations are significant, with the only exception being the correlation between milk and hair for I, Mo and Co, and for Co between blood serum and milk and whole-blood and milk. Only in the case of Se, a significant correlation was observed between hair and milk (*r* = 0.6612). In the case of Co, Cu and Se, the concentrations found in hair were significantly correlated with the levels of the same elements in blood serum or whole blood, but not for Mo and I. These results would suggest that hair cannot be considered a good indicator of the elemental status in cattle. This seems also to corroborate previous studies by Roug et al. in deer [24], and Grabyszuk et al., who questioned the suitability of hair samples for elemental analysis in cattle [25]. Significant correlations were observed between the levels found in milk and blood serum for all elements, with the only exception of Co. In the case of Mo and Se, these correlations were strong, with *r* > 0.8. The poor correlation between Co in milk and blood serum found in this study would require further investigation. Previous studies by Akins et al. observed that while the addition of Co to the diet did not affect plasma vitamin B12 concentrations, it did increase vitamin B12 concentration in milk [26]. This may suggest that the Co concentration in blood may be regulated by homeostatic mechanisms. Although in the present work, a significant correlation was found between the levels of Cu in milk and blood serum (*r* = 0.676); Wang et al. found no correlation between Cu in the same matrices (*r* = 0.013, *P* = 0.933) and concluded that mammary glands control the concentrations of milk Cu to prevent maternal deficiency or excess [27]. Therefore, the content of Cu in milk may not be suitable to reflect the status of this nutrient in serum [27]. This seems to contradict the results obtained in the present study for Cu. However, it must be noted that for all specimens in this study, the levels of Cu were adequate, and therefore, further investigations with cows at different Cu-deficiency status would be necessary to corroborate the hypothesis of Wang et al. for the homeostatic control of Cu by the mammary glands [27].

**Table 2:** Pearson correlations for the contents of Co, Cu, Se, Mo and the four types of biomarkers samples (*n* indicated the number of observations)

Biomarkers	Co		Cu		Se		Mo		I	
	***r***	***n***	***r***	***n***	***r***	***n***	***r***	***n***	***r***	***n***
Blood serum—hair	0.6699*	119	0.7452*	119	0.6653*	119	0.5217*	119	0.6351*	119
Blood serum—milk	0.2563	72	0.6760*	72	0.8774*	72	0.8526*	72	0.6499*	72
Whole blood—hair	0.6071*	119	0.7664*	119	0.7080*	119	0.5333*	119	0.5456*	119
Whole blood—milk	0.1794	69	0.6898*	69	0.5480*	69	0.5712*	69	0.5396*	69
Whole blood—blood serum	0.5962*	119	0.8573*	119	0.7740*	119	0.7957*	119	0.8317*	119
Milk—hair	0.0361	69	0.5268*	69	0.6612*	69	0.3041	69	0.2472	69

^*^Significant at *P* ≤ 0.01

Significant correlations were observed between the levels in blood serum and whole blood in the case of Cu, Se, Mo and I (*r* > 0.8) and Co (*r* ~ 0.6); however, a paired *t*-test [23] showed that for all elements, there is a significant difference (95% confidence) between the levels in blood serum and whole blood, suggesting differences in the partition of the elements in blood [28–30], while for I and Cu, the levels in whole blood were higher than in serum. The opposite trend was observed for Co, Mo and Se. Luna et al. investigated the concentration of essential and toxic elements in the serum and plasma of cattle, observing that Cu and Se levels were lower in the serum than in plasma, and there were no significant differences for the other elements, including Co and Mo [31]. The disagreement between the studies highlights the difficulty of assessing the elemental status using blood serum, as it requires rigorous control experimental conditions to avoid cellular rupture, and therefore, it may be of more difficult application in remote locations and on-field studies. The analysis of whole blood would seem the most robust approach for this type of investigation.

### Mineral Composition of Cow Feed: Forage, Concentrates and Nutritional Supplements

The summary data of the elemental composition of forage, concentrates and nutritional supplements can be found in Tables S9–11, while the results for Co, Cu, Se and Mo are represented as box plots in Fig. 2i and j.

The National Research Council (NRC, US) minimum dietary requirement for Co is 0.11 mg/kg in total diet for maintaining vitamin B12 levels [8]. The concentrations of Co found in concentrates were ≤ 0.35–13.4 mg/kg d.w. (dry weight) and ≤ 0.35–207 mg/kg d.w. in nutritional supplements. Levels of 0.25 to 0.35 mg/kg have been shown to improve production parameters independent of vitamin B12 levels [32]. Toxic levels are > 30 mg/kg and were exceedingly rare, and these were only found in limestone powder provided as nutritional supplements (61.4 ± 62.8 mg/kg d.w., *n* = 9, Table S11). Most of the rice-based feeds were low in cobalt (≤ 0.35–1.05 mg/kg d.w., Table S10). Black pigweed or *Trianthema portulacastrum* (Sarana, ≤ 0.35–5.19 mg/kg d.w. Co) and CO3 grass (≤ 0.35–5.06 mg/kg d.w. Co) were generally low in Co, whereas ipil-ipil (*Leucaena leucocephala*) was generally adequate in Co (1.26–5.09 mg/kg d.w. Co, Table S9). *Azolla*, a high-protein aquatic fern commonly used as a supplement in the dairy industry to help meet protein requirements displayed the highest concentrations of cobalt amongst the local forage (as well as Zn, Fe and Mn, Table S9) in the range between 1.17 and 13.4 mg/kg d.w. Reports suggest that this trend is reciprocated worldwide; due to the high potential of aquatic plants to accumulate trace elements [33]. Even though most fodders reached, or even exceeded, the minimum NRC requirement for dietary Co, all the cows included in this study were deficient in Co (see the “Elemental Deficiency of Crossbred Cows: Analysis of Whole Blood, Serum, Hair and Milk” section); therefore, some antagonistic effects or limited bioavailability of the Co present in the feed would explain the Co deficiency of the cows bred in the studied areas. Cobalt levels in crops and forages can be very geographically specific, depending on the underlying geology of the soil and interactions in the soil. Alkaline pH and the presence of Mn mean that Co is unavailable to plants. Hence, neighbouring fields may have different availability of Co for plants and the animals that graze them. Cattle are also known to ingest soil (a behaviour known as *pica* [34]) and thus, the levels in animals may not reflect the measured intake in feed. As can be observed in Fig. 2e, there were no specific regional differences for Co levels in the local forage, but variations within the different regions.

With regard to Cu, the concentration in local forages was in the ranges of ≤ 0.7 (LOD) and 202 mg/kg d.w., ≤ 0.7–43.8 mg/kg d.w. in concentrates, and 12.0–3975 mg/kg d.w. in nutritional supplements, the highest values observed for limestone-based supplements (2426 ± 1295 mg/kg d.w. Cu, *n* = 9, Table S11). The toxic level of Cu according to the NRC (US) is > 40 mg/kg d.w. feed [8]. However, this is for the American context with antagonists present and causing chronic toxicity and an acute haemolytic crisis. A few fodders (CO3, *Gliricidia* and ipil-ipil, Table S9), as well as nutritional supplements (Table S11), had concentrations approaching or above this toxic level. This, however, would be diluted out by other feeds. The median Cu concentrations across all feed samples (median = 3.25–19.9 mg/kg d.w. Cu, Table S9) and supplement samples (median = 3.92–18.0 mg/kg d.w. Cu, Table S10) were consistent and in agreement with previously recorded values, while exceeding the concentrations reported in infertile regions [35].

The NRC minimum dietary Mo requirement is around 15 mg/kg for adult lactating cattle depending on the level of antagonists [8]. The level of Mo in commonly utilised feeds was low; below the NRC 10 mg/kg where it can interfere with copper absorption. The concentration in local forages ranged in the interval of ≤ 0.75 (LOD) and 4.96 mg/kg d.w., ≤ 0.75–3.41 mg/kg d.w. in concentrates and from ≤ 0.75 to 3.18 mg/kg d.w. in nutritional supplements, with beer waste the only feed with all values above the limits of detection (Table S10). Most forages commonly utilised in the study areas were below the 15 mg/kg NRC requirement level (Table S9). While Mo is an essential element present in some enzymes, its requirement is very small making deficiency very rare, and it is usually measured as it is an antagonist for Cu absorption. The low levels of Mo observed in this study would not limit Cu absorption. While this may be beneficial for some low-Cu fodders, this means that the risk of Cu toxicity needs to be considered when using supplements, as the cows had adequate Cu in blood serum (Table 1 and Table S6).

The NRC minimum dietary Se requirement is around 0.3 mg/kg for adult lactating cattle [8]. Chronic toxicity can occur when cattle are fed diets with 5 to 40 mg/kg Se for a period of several weeks or months. The levels of Se in local forage ranged over the interval between ≤ 0.30 (LOD) and 9.74 mg/kg d.w. (Table S9), ≤ 0.30–1.11 mg/kg d.w. in concentrates (Table S10) and 0.32–108 mg/kg d.w. in nutritional supplements (Table S11). Most of the commonly utilised fodders in the study area were around the dietary requirements except for most of the rice/paddy products.

In terms of any regional variations for local forage (Fig. 2i and j), there was a high degree of consistency across all regions when comparing Co, Cu and Se. As grazing nutrition in Sri Lanka is largely based upon seasonal availability, it is difficult to select which variety of fodder should form the base diet. However, it is worth noting that Sarana grass offers a good elemental profile.

### Principal Components Analysis

#### Elemental Concentrations in Biomarkers

**Fig. 3:**
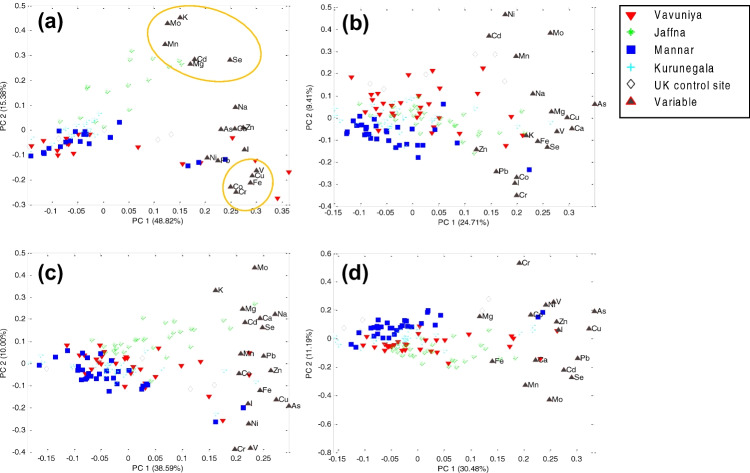
Principal component analysis (PCA) biplot of the biological samples and element variables for the first two principal components. The variance explained by each PC appears indicated on the axes. PCA models for **a** milk samples, **b** hair, **c** blood serum and **d** whole blood

In relation to all the biomarker samples, the PCA model for milk (matrix dimension 78 × 18) is the one with the highest percentage of explained variance: 70.35% variance for 3 PCs. The highest positive correlation was observed for a group of elements including Co, Cr, Fe, Cu and V (Fig. 3a), which appeared at high loading values of PC1. Although the complete geographical classification of the milk samples was not possible, it is quite clear that all samples from Jaffna, on the North coast of Sri Lanka, tend to separate from the group and showed higher levels of K, Mo, Mn, Mg, Cd and Se, which appeared associated at high loadings of PC1 and PC2. It is worth noting that the milk samples from the control site in the UK overlap with the majority and follow the general trend.

The Pearson correlation between the elemental concentrations in hair samples is rather poor, as anticipated earlier in the “Elemental Deficiency of Crossbred Cows: Analysis of Whole Blood, Serum, Hair and Milk” section, and it can be confirmed by the low variance explained by the PCA models (126 × 18, 41.72% for 3 PCs). Consequently, it is not possible to observe any grouping of the variables (Fig. 3b) or separation of samples according to geographical origin. However, there is an incipient grouping of samples from Vavuniya, at higher loading of PC1 and PC2, and from Mannar at the lowest loading of both PCs.

The PCA model for blood serum (127 × 18) and whole blood samples (127 × 18), explained moderate percentages of variance in the samples for models with 3 PCs, of 55.60% and 49.76%, respectively. As in the case of the milk samples, the samples from Jaffna separate quite clearly from most of the samples (Fig. 3c, d), which also overlap with the control samples from the UK. Although not as clear as the milk samples, it is possible to appreciate a similar grouping of the elements with K, Mo, Mg, Cd, Ca and Se also at high loadings of PC1 for blood serum (Fig. 3c), mirroring the behaviour of the samples from Jaffna. The samples from Mannar and Kurunegala seemed to be displaced to the lower scores of PC1 and PC2, indicating lower levels for all elements.

#### Elemental Concentrations in Feed

**Fig. 4:**
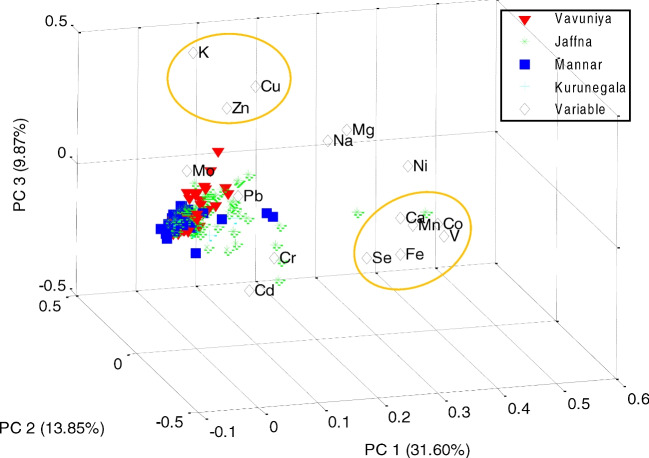
Principal component analysis (PCA) biplot of the forage samples and element variables for the first three principal components. The variance explained by each PC appears indicated on the axes

In the first instance, PCA modelling was considered for the local forage samples only (169 × 16), to reveal any significant differences in the elemental composition between the forage obtained from the four geographical regions. A 3-PC model explained 55.31% of the sample variance. The plotting of the loading of the 3 PCs (Fig. 4) showed some grouping of the elements (K, Zn and Cu) at high loadings of PC2 and PC3 and negative loadings of PC1. Other groupings of elements, including Ca Mn, Co, V, Fe and Se, can be found at high loading of PC1. This is similar to the association of elements at high loadings of PC1 in the model for milk samples (Fig. 3a). However, the grouping of the other elements differs. This would suggest that other factors, such as availability or antagonistic effects, may affect the concentration in milk, other than the total element concentration in the feed. Although separation according to the geographic region is not complete, some incipient grouping can be observed, and as for the animal samples, the lowest scores were observed for the Mannar and Kurunegala samples, while samples from Vavuniya tend to spread towards the group of elements formed by K, Cu and Zn, and the samples from Jaffna are orientated towards the group of non-essential/toxic elements (Fig. 4). The same set of scores was plotted to try to highlight the different species of local fodder, but the separation was more difficult (Figure S1 in SI). Still, paddy straw samples seem to gather towards the corner of the new 3-PC space, with the lowest scores for all three principal components.

**Fig. 5:**
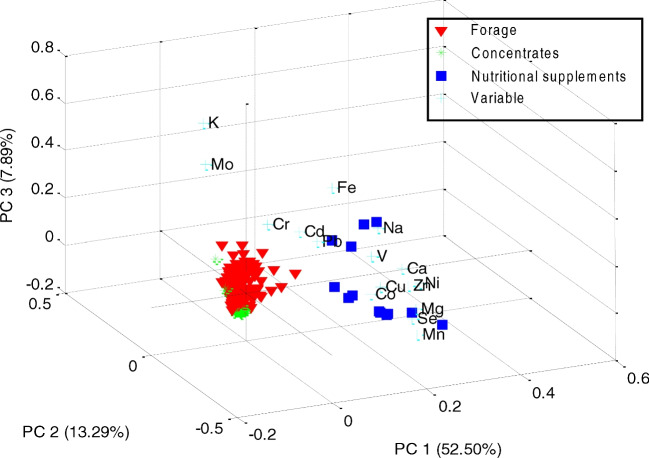
Principal components analysis (PCA) biplot of the feed samples (forage, concentrates and nutritional supplements) and element variables for the first three principal components. The variance explained by each PC appears indicated on the axes

When all three types of livestock feed (forage, concentrates and nutritional supplements) were included in the modelling (249 × 16 matrix), the variance explained by a 3-PC model was much higher (73.68%) than for the forage only (57.31%). The incipient geographical separation observed for the samples of forage (Fig. 4) was lost by including concentrates and nutritional supplement samples in the dataset, since feed supplements may have a different geographical origin to the point of consumption. However, when the scores of the samples were plotted to highlight the three types of feed, the separation according to the elemental composition becomes very clear (Fig. 5), with the nutritional supplement samples showing the highest concentration of most elements, situated at high scores of PC1. Interestingly, some of the nutritional supplements spread as well towards high loadings of PC2, suggesting the predominance of non-essential or even toxic elements, such as Cd and Pb in this kind of sample over the other two types of feeds. This observation agrees also with the conclusions by Orjales et al., who reported higher concentrations of toxic elements (mainly As, Cd and Pb) as well as Cr and Ni in mineral supplements and therefore recommended more strict monitoring of these supplements when used for animal nutrition [36]. Overall, the lowest scores appear to be associated with the concentrates, overlapping partially with some of the forages. This could be due to the abundance of rice by-products amongst this type of feed, which showed the lowest elemental concentrations. It also suggests that although some of the concentrates could meet the dietary intake requirements for certain elements, they alone cannot provide a complete and balanced diet; therefore, their use as the main nutritional source is discouraged.

## Conclusions

The cross-breed cows in all four areas of Sri Lanka were deficient in I and Co and additional dietary supplements would be beneficial. The level of Cu was generally low in the fodder materials, but adequate in the cows suggesting that the Cu is easily absorbed. Evaluation of the concentration of Co, Cu, Se, Mo and I in hair showed a very poor correlation with the levels of milk and blood, confirming that hair samples are poor indicators of the mineral or elemental status of cattle. Most of the local forages analysed meet dietary requirements, with the exception of rice/paddy products. Black pigweed or *Trianthema portulacastrum* (Sarana) grass offers the best elemental profile of all the forage samples investigated in this study. The analysis of concentrates and mineral supplements revealed that whilst they may provide required levels for certain elements, the levels were variable and in some cases, did not correlate with stated levels of trace elements.

## Supplementary Information

Below is the link to the electronic supplementary material.

Supplementary file1 (DOCX 239 KB)

## Data Availability

Data is provided within the manuscript or supplementary information files.
