# Supplementary material for: Assessment of Elemental Deficiency of Crossbred Dairy Cows and Mineral Composition in Natural Feed and Nutritional Supplements in the Northern and Northwestern Provinces in Sri Lanka
Source: Biol Trace Elem Res. 2024 Jul 12;203(4):2029–40. doi: 10.1007/s12011-024-04299-x (PMC11919970; doi:10.1007/s12011-024-04299-x)
Supplement: Supplementary file 1 — Supplementary file1 (DOCX 239 KB) [file 12011_2024_4299_MOESM1_ESM.docx]

**Supplementary Information**

**Assessment of the elemental deficiency of crossbred dairy cows and mineral composition in natural feed and nutritional supplements in the Northern and North Western provinces in Sri Lanka**

*Joseph Clarkson^a^*, Neil I Ward^a^, Joaquín M. Prada^b^, David Tisdall^b^, Mónica Felipe-Sotelo^a¥^, Janak Vidanarachchi^c^, Mike Christian^d^ Mark Chambers^e^.*


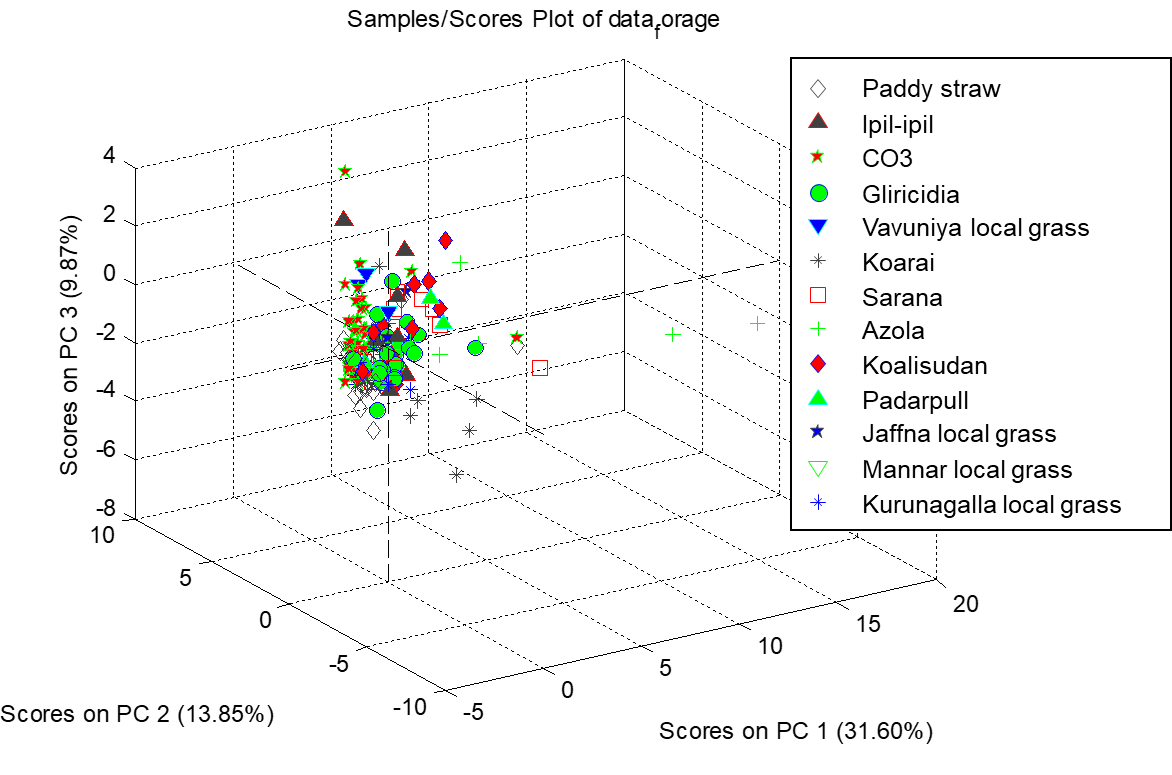


**Figure S1.-** Principal component analysis (PCA) plot for the forage samples representing scores for the first three principal components. Samples have been labelled according to the type of forage and the variance explained by each PC appears indicated on the axes.

**Table S1-** Summary of information, body condition scores (BCS) and fertility parameters for the cows included in the study.

| **Location** | **Cow ID** | **Breed** | **Colour** | **BCS** | **Fertility parameters** | | | | | **Mineral Supplementation** | |
| --- | --- | --- | --- | --- | --- | --- | --- | --- | --- | --- | --- |
|  |  |  |  |  | **Age at sampling /  year of birth** | **Year of  1^st^ calving** | **Number of  calvings** | **Pregnancy  Status  Yes/No** | **Number of  services current pregnancy** | **Yes/No** | **Brand** |
| Vavuniya | VAV01 | Jersey / Local | Brown | 3 | 6 / 2012 | 2014 | 6 | Yes | 1 | N/A | N/A |
|  | VAV02 | Sahiwal / Local | Brown | 2.5 | 7 / 2011 | 2013 | 4 | Yes | 1 | N/A | N/A |
|  | VAV03 | Jersey / Local | Dark brown | 2.75 | 4 / 2015 | 2016 | 4 | No | N/A | Yes | Super Mineral Mix |
|  | VAV04 | Jersey / Local | Brown | 2.5 | 8 / 2010 | 2012 | 3 | Yes | 1 | Yes | Super Mineral Mix |
|  | VAV05 | Jersey / Local | Brown | 3 | 7 / 2011 | 2014 | 2 | Yes | 1 | Yes | Fertigen |
|  | VAV06 | Jersey / Local | Brown | 2.75 | 2 / 2016 | 2018 | 1 | Yes | 1 | Yes | Fertigen |
|  | VAV07 | Jersey / Local | Black & white | 2.5 | 4 / 2015 | 2017 | 2 | Yes | 1 | Yes | Super Mineral Mix |
|  | VAV08 | Jersey / Local | Brown | 3.25 | 6 / 2012 | 2015 | 2 | Yes | 1 | Yes | Super Mineral Mix |
|  | VAV09 | Jersey / Local | Brown | 3 | 2 / 2016 | 2018 | 1 | Yes | 1 | N/A | N/A |
|  | VAV10 | Jersey / Local | Brown | 2.5 | 6 / 2012 | 2015 | 1 | Yes | 1 | N/A | N/A |
|  | VAV11 | Jersey / Local | Black | 3.5 | 3 / 2016 | 2018 | 1 | Yes | 1 | N/A | N/A |
|  | VAV12 | Sahiwal / Local | Brown | 2.75 | 5 / 2014 | 2017 | 1 | Yes | 1 | N/A | N/A |
|  | VAV13 | Jersey / Local | Light brown | 3 | 4 / 2015 | 2017 | 2 | Yes | 1 | N/A | N/A |
|  | VAV14 | Jersey/ Local | Brown | 3 | 5 / 2014 | 2017 | 1 | Yes | 1 | N/A | N/A |
|  | VAV15 | Jersey / Local | Light brown | 2.5 | 4 / 2015 | 2017 | 3 | Yes | 1 | Yes | Super Mineral Mix |
|  | VAV16 | Jersey / Local | Light Brown | 2.25 | 8 / 2010 | 2013 | 3 | Yes | 1 | Yes | Super Mineral Mix |
|  | VAV17 | Jersey / Local | Dark brown | 2.75 | 2 / 2017 | N/A | 1 | No | N/A | Yes | Fertigen |
|  | VAV18 | Jersey / Local | dark brown | 2.25 | 8 / 2010 | 2013 | 1 | Yes | 1 | Yes | Fertigen |
|  | VAV19 | Jersey / Local | Brown | 2.75 | 5 / 2014 | 2016 | 3 | No | N/A | N/A | N/A |
|  | VAV20 | Jersey / Local | Brown & white | 2.75 | 7 / 2011 | 2014 | 3 | Yes | 1 | N/A | N/A |
|  | VAV21 | Jersey / Local | Light brown | 3.25 | 3 / 2016 | 2018 | 2 | Yes | 1 | Yes | Fertigen |
|  | VAV22 | Jersey / Local | Light brown | 2.5 | 5 / 2014 | 2015 | 2 | Yes | 1 | Yes | Fertigen |
|  | VAV23 | Jersey / Local | Dark brown | 2.25 | 5 / 2014 | 2015 | 3 | Yes | 1 | Yes | Fertigen |
|  | VAV24 | Jersey / Local | Dark brown | 3.25 | 8 / 2010 | 2013 | 1 | Yes | 1 | Yes | Fertigen |
|  | VAV25 | Jersey / Local | White & black | 3 | 7 / 2011 | 2014 | 1 | Yes | 1 | N/A | N/A |
|  | VAV26 | Sahiwal / Local | Brown | 2.5 | 8 / 2010 | 2013 | 3 | Yes | 1 | N/A | N/A |
|  | VAV27 | Jersey / Local | Brown & white | 2.25 | 2 / 2016 | 2018 | 1 | Yes | 1 | N/A | N/A |
|  | VAV28 | Sahiwal / Local | Brown | 3.5 | 4 / 2015 | N/A | N/A | Yes | 1 | N/A | N/A |
|  | VAV29 | Jersey / Local | Brown | 3.5 | 3 / 2016 | 2018 | 2 | Yes | 1 | N/A | N/A |
|  | VAV30 | Jersey / Local | Dark brown | 3.25 | 6 / 2015 | N/A | N/A | Yes | 1 | N/A | N/A |

**Table S1-** Continuation

| **Location** | **Cow ID** | **Breed** | **Colour** | **BCS** | **Fertility parameters** | | | | | **Mineral Supplementation** | |
| --- | --- | --- | --- | --- | --- | --- | --- | --- | --- | --- | --- |
|  |  |  |  |  | **Age at sampling /  year of birth** | **Year of  1^st^ calving** | **Number of  calvings** | **Pregnancy  Status  Yes/No** | **Number of  services current pregnancy** | **Yes/No** | **Brand** |
| Jaffna | JAF01 | Jersey / Local | Light brown | 2.75 | 5 / 2013 | N/A | 3 | No | N/A | Yes | Minamax |
|  | JAF02 | Jersey / Local | Light brown | 2.5 | 3 / 2015 | N/A | N/A | Yes | 1 | Yes | Minamax |
|  | JAF03 | Jersey / Local | Brown | 2.5 | 7 / 2011 | N/A | 3 | No | N/A | Yes | Minamax |
|  | JAF04 | Jersey / Local | Brown | 3 | 7.5 / 2010 | N/A | 2 | No | N/A | Yes | Minamax |
|  | JAF05 | Jersey / Local | Dark brown | 3 | 2.25 / 2016 | N/A | N/A | No | N/A | Yes | Minamax |
|  | JAF06 | Jersey / Local | Black & brown | 3.25 | 7.5 / 2011 | N/A | 2 | Yes | 2 | Yes | Minamax |
|  | JAF07 | Jersey / Local | Brown | - | 5.25 / 2013 | N/A | 3 | Yes | 1 | Yes | Nutrisacc |
|  | JAF08 | Jersey / Local | Light brown | 2.75 | 03 / 2015 | 2017 | 1 | No | N/A | Yes | Nutrisacc |
|  | JAF09 | Jersey / Local | Brown |  | 7 / 2011 | N/A | 3 | No | N/A | Yes | Minamax |
|  | JAF10 | Jersey / Local | Light brown | 2.5 | 7 / 2011 | N/A | 2 | No | N/A | Yes | Nutrisacc |
|  | JAF11 | Jersey / Local | White | 2.25 | 6.5 / 2012 | N/A | 3 | Yes | 1 | Yes | Minamax |
|  | JAF12 | Jersey / Local | Brown | 2.75 | 2.5 / 2015 | N/A | N/A | No | N/A | Yes | Minamax |
|  | JAF13 | Jersey / Local | Light brown | 2.75 | 06 / 2012 | N/A | 3 | No | N/A | Yes | Aminol |
|  | JAF14 | Jersey / Local | Dark brown | 2.25 | 3 / 2015 | N/A | N/A | Yes | 1 | Yes | Aminol |
|  | JAF15 | Jersey / Local | Brown | 3.5 | 7 / 2011 | N/A | 1 | Yes | 1 | Yes | Aminol |
|  | JAF16 | Jersey / Local | Light brown | 3.25 | 6 / 2012 | N/A | 1 | No | N/A | Yes | Nutrisacc |
|  | JAF17 | Jersey / Local | Brown & white | 2.75 | 5 / 2013 | 2016 | 2 | No | N/A | Yes | Nutrisacc |
|  | JAF18 | Jersey / Local | Dark brown | 2.75 | 7 / 2011 | N/A | 2 | No | N/A | Yes | Super Mineral Mix |
|  | JAF19 | Jersey / Local | Light brown | 2.5 | 6.5 / 2012 | N/A | 3 | No | N/A | Yes | Super Mineral Mix |
|  | JAF20 | Jersey / Local | Brown & black | 3 | 2.25 / 2016 | N/A | N/A | No | N/A | Yes | Super Mineral Mix |
|  | JAF21 | Jersey / Local | Brown | 2.75 | 7 / 2011 | N/A | 2 | No | N/A | Yes | Nutrisacc |
|  | JAF22 | Jersey / Local | Light brown | 3.25 | 2.5 / 2016 | N/A | N/A | No | N/A | Yes | Nutrisacc |
|  | JAF23 | Jersey / Local | Light brown | 2.75 | 5.5 / 2013 | N/A | 3 | No | N/A | Yes | Nutrisacc |
|  | JAF24 | Jersey / Local | Brown | 3 | 5 / 2013 | N/A | 2 | No | 2 | Yes | Aminol |
|  | JAF25 | Jersey / Local | Light brown | 3.25 | 2.5 / 2016 | N/A | N/A | Yes | 5 | Yes | Aminol |
|  | JAF26 | Jersey / Local | Brown & black | 3.25 | 4 / 2014 | N/A | 1 | No | N/A | Yes | Minamax |
|  | JAF27 | Jersey / Local | Brown | 2.5 | 3.5 / 2015 | 2017 | 1 | Yes | 4 | Yes | Super Mineral Mix |
|  | JAF28 | Jersey / Local | Brown | 2.75 | 4.25 / 2014 | 2014 | 2 | Yes | 1 | Yes | Super Mineral Mix |
|  | JAF29 | Jersey / Local | Black | 2.75 | 3.75 / 2014 | 2017 | 1 | No | 3 | Yes | Super Mineral Mix |
|  | JAF30 | Jersey / Local | Brown & black | 3.25 | 3.75 / 2014 | 2017 | 1 | Yes | 1 | Yes | Super Mineral Mix |

**Table S1-** Continuation

| **Location** | **Cow ID** | **Breed** | **Colour** | **BCS** | **Fertility parameters** | | | | | **Mineral Supplementation** | |
| --- | --- | --- | --- | --- | --- | --- | --- | --- | --- | --- | --- |
|  |  |  |  |  | **Age at sampling /  year of birth** | **Year of  1^st^ calving** | **Number of  calvings** | **Pregnancy  Status  Yes/No** | **Number of  services current pregnancy** | **Yes/No** | **Brand** |
| Mannar | MAN01 | Sahiwal / Local | Brown | 2.75 | 6 / 2012 | 2014 | 3 | Yes | 2 | Yes | Super Mineral mix |
|  | MAN02 | Jersey / Local | White | 2.25 | 4 / 2014 | N/A | 1 | Yes | N/A | Yes | Super Mineral mix |
|  | MAN03 | Jersey / Local | Brown | 3.5 | 3 / 2014 | N/A | N/A | Yes | N/A | Yes | Vitamin Mineral mix |
|  | MAN04 | Jersey / Local | Brown | 3.5 | 2.5 / 2015 | N/A | N/A | Yes | N/A | Yes | Vitamin Mineral mix |
|  | MAN05 | Jersey / Local | Pure white | 2.5 | 6 / 2012 | N/A | 1 | Yes | 1 | Yes | Vitamin Mineral mix |
|  | MAN06 | Jersey / Local | Dark white | 3 | 6 / 2012 | 2014 | 5 | No | 3 | Yes | Vitamin Mineral mix |
|  | MAN07 | Jersey / Local | Pure white | 2.5 | 5 / 2013 | N/A | 1 | Yes | 1 | Yes | Vitamin Mineral mix |
|  | MAN08 | Sahiwal / Local | Brown | 2.25 | 4 / 2014 | 2016 | 2 | Yes | 1 | Yes | Vitamin Mineral mix |
|  | MAN09 | Sahiwal / Local | Black & brown | 2.5 | 6 / 2012 | 2017 | 2 | Yes | 1 | Yes | Fertigen |
|  | MAN10 | Sahiwal / Local | Brown | 2.5 | 10 / 2008 | 2013 | 5 | No | N/A | Yes | Vitamin Mineral mix |
|  | MAN11 | Sahiwal / Local | Light brown | 2.75 | 4 / 2014 | 2014 | 1 | No | N/A | Yes | Vitamin Mineral mix |
|  | MAN12 | Jersey / Local | Brown | 2.5 | 4 / 2014 | 2017 | 2 | Yes | 1 | Yes | Vitamin Mineral mix |
|  | MAN13 | Sahiwal / Local | Brown | 2.5 | 5 / 2013 | 2015 | 3 | Yes | 2 | Yes | Super Mineral mix |
|  | MAN14 | Jersey / Local | White & brown | 3.25 | 4 / 2014 | N/A | N/A | Yes | 1 | Yes | Super Mineral mix |
|  | MAN15 | Jersey / Local | Brown | 2.5 | 8 / 2010 | 2013 | 5 | Yes | N/A | Yes | Super Mineral mix |
|  | MAN16 | Jersey / Local | White | 2.5 | 6 / 2012 | 2015 | 3 | No | N/A | Yes | Super Mineral mix |
|  | MAN17 | Jersey / Local | Black & brown | 2.5 | 6 / 2012 | 2017 | 2 | No | N/A | Yes | Super Mineral mix |
|  | MAN18 | Jersey / Local | Black & white | 2.5 | 4 / 2014 | 2017 | 1 | Yes | 1 | Yes | Super Mineral mix |
|  | MAN19 | Jersey / Local | White | 2.5 | 6 / 2012 | N/A | N/A | N/A | 2 | Yes | Vitamin Mineral mix |
|  | MAN20 | Jersey / Local | White | 3 | 7 / 2011 | 2014 | 5 | Yes | 1 | Yes | Super Mineral mix |
|  | MAN21 | Jersey / Local | Brown | 2.75 | 6 / 2012 | 2015 | 4 | Yes | N/A | Yes | Super Mineral mix |
|  | MAN22 | Sahiwal / Local | Brown & black | N/A | 3 / 2015 | 2018 | 1 | Yes | 1 | Yes | Vitamin Mineral mix |
|  | MAN23 | Jersey / Local | Light Brown | 2.5 | 6 / 2012 | N/A | N/A | No | N/A | Yes | Vitamin Mineral mix |
|  | MAN24 | Sahiwal / Local | Brown, black & white | 2.75 | 5 / 2013 | 2014 | 3 | No | N/A | Yes | Vitamin Mineral mix |
|  | MAN25 | Jersey / Local | Black & white | 2.75 | 3 / 2015 | 2017 | 2 | Yes | 1 | Yes | Vitamin Mineral mix |
|  | MAN26 | Sahiwal / Local | Brown | 2.25 | 6 / 2012 | N/A | 1 | No | N/A | Yes | Vitamin Mineral mix |
|  | MAN27 | Jersey / Local | Brown | 2.5 | 3 / 2015 | 2017 | 2 | No | N/A | Yes | Vitamin Mineral Mix |
|  | MAN28 | Jersey / Local | Black & brown | 2.25 | 5 / 2013 | N/A | 3 | No | N/A | Yes | Fertigen |
|  | MAN29 | Jersey / Local | Light brown | 2.75 | 3.5 / 2014 | N/A | N/A | Yes | N/A | Yes | Super Mineral mix |
|  | MAN30 | Jersey / Local | Brown | 2.5 | 5 / 2013 | 2016 | 2 | Yes | N/A | Yes | Super Mineral mix |

**Table S1-** Continuation

| **Location** | **Cow ID** | **Breed** | **Colour** | **BCS** | **Fertility parameters** | | | | | **Mineral Supplementation** | |
| --- | --- | --- | --- | --- | --- | --- | --- | --- | --- | --- | --- |
|  |  |  |  |  | **Age at sampling /  year of birth** | **Year of  1^st^ calving** | **Number of  calvings** | **Pregnancy  Status  Yes/No** | **Number of  services current pregnancy** | **Yes/No** | **Brand** |
| Kurungulla | KUR01 | Jersey / Local | Light brown | 2.5 | 3 /2015 | 2018 | 1 | No | N/A | Yes | Proban Basic |
|  | KUR02 | Jersey / Local | Brown | 2.25 | 8 / 2010 | N/A | 5 | No | N/A | Yes | Proban Basic |
|  | KUR03 | Jersey / Local | Brown | 2.5 | 5 / 2013 | 2015 | 3 | No | N/A | Yes | Proban Basic |
|  | KUR04 | Jersey / Local | Brown | 2.25 | 3 /2015 | 2015 | 2 | No | N/A | Yes | Proban Basic |
|  | KUR05 | Jersey / Ayrshire | Light brown | 2.5 | 4 /2014 | 2015 | 2 | No | 6 | Yes | Proban Basic |
|  | KUR06 | Jersey / Local | Light brown | 3.5 | 5 / 2013 | 2017 | 1 | Yes | 5 | Yes | Aminol |
|  | KUR07 | Friesian / Sahiwal | Black | 2.75 | 7 /2011 | N/A | 5 | No | N/A | Yes | Aminol |
|  | KUR08 | Jersey / Friesian | Black & white | 2.25 | 3 /2015 | 2018 | 1 | Yes | 1 | Yes | Aminol |
|  | KUR09 | Jersey / Friesian | Light brown | 3 | 3 /2015 | 2018 | 1 | Yes | 1 | Yes | Aminol |
|  | KUR10 | Jersey / Sahiwal | Brown | 3.25 | N/A | N/A | 5 | No | 3 | Yes | Aminol |
|  | KUR11 | Friesian / Local | Black | 3.25 | 4 /2014 | N/A | 5 | No | 8 | Yes | Aminol |
|  | KUR12 | Jersey / Friesian | Brown | 2.5 | 4 /2014 | 2017 | 1 | No | 3 | Yes | Aminol |
|  | KUR13 | Jersey / Local | Brown | 2.5 | 5 / 2013 | 2013 | 4 | No | 12 | Yes | Proban Basic |
|  | KUR14 | Jersey / Sahiwal | Brown | 2.75 | 3 /2015 | 2017 | 1 | No | 6 | Yes | Proban Basic |
|  | KUR15 | Friesian / Sahiwal | Black & white | 3 | 3 /2015 | 2017 | 1 | No | 5 | Yes | Proban Basic |
|  | KUR16 | Friesian / Sahiwal | Black & white | 3.25 | 5 / 2013 | N/A | 3 | Yes | 4 | Yes | Aminol |
|  | KUR17 | Ayrshire / Local | Brown & white | 3 | 3 /2015 | N/A | N/A | No | 4 | Yes | Aminol |
|  | KUR18 | Jersey / Friesian | Black & white | 2.75 | 4 /2014 | N/A | 2 | Yes | 2 | Yes | Aminol |
|  | KUR19 | Jersey / Sahiwal | Brown | 2.75 | 5 / 2013 | N/A | 3 | No | 3 | Yes | Aminol |
|  | KUR20 | Jersey / Friesian | Light brown | 2.5 | 3 /2015 | 2018 | 1 | No | 1 | Yes | Proban Basic |
|  | KUR21 | Jersey / Local | Brown | 2.75 | 6 /2012 | 2016 | 4 | No | N/A | Yes | Proban Basic |
|  | KUR22 | Jersey / Local | Brown | 2.25 | 5 / 2013 | N/A | 3 | No | N/A | Yes | Proban Basic |
|  | KUR23 | Jersey / Friesian | Black | 2 | 5 / 2013 | N/A | 3 | No | N/A | Yes | Proban Basic |
|  | KUR24 | Jersey / Local | Light brown | 3 | 5 / 2013 | 2012 | 4 | No | 6 | Yes | Aminol |
|  | KUR25 | Jersey / Local | Black & brown | 3 | 2 / 2016 | N/A | N/A | No | 2 | Yes | Proban Basic |
|  | KUR26 | Jersey / Local | Brown | 2.75 | N/A | N/A | 5 | N/A | 6 | Yes | Proban Basic |
|  | KUR27 | Jersey / Local | Black | 2.75 | N/A | 2015 | 2 | No | 3 | Yes | Proban Basic |
|  | KUR28 | Jersey / Sahiwal | Light brown | 2.5 | N/A | 2016 | 2 | No | N/A | Yes | Proban Basic |
|  | KUR29 | Jersey / Local | Light brown | 2.75 | N/A | 2016 | 1 | Yes | N/A | Yes | Aminol |
|  | KUR30 | Jersey / Sahiwal | Brown | 3.25 | N/A | 2017 | N/A | No | 6 | Yes | Aminol |

**Table S2-** Detailed information of the local fodder and concentrates used by local dairy farmers in the sampling area
(Vavuniya, Mannar, Jaffna and Kurunegala, in Sri Lanka).

| **Forages** | | | |
| --- | --- | --- | --- |
| **Sample** | **English common name** | **Local name** | **Comments** |
| *Azolla pinnata* | Azolla | - | An aquatic fern grown in tanks |
| *Pennisetum perpureum* X *Pennisetum americarnum* | CO-3/CO-4 | - | Various trade names and hybrid varieties for improved hybrids of Napier grass. But these were all grouped under CO-3. |
| *Gliricidia sepium* | Gliricidia | - | A leguminous tree |
| Leucaena leucocephala | Ipil Ipil | - | A leguminous tree. |
| *Cyperus corymbosus* | - | Local Sinhala name is “*Gal ehi*”. Local Tamil name “*Korai*” (கோரை). | A native tropical plant |
| *Trianthema portulacastrum* | Black pigweed | Local Sinhala name is “Sarana”. | - |
| *Oryza sativa* | Paddy straw | - | Straw by-product of rice production |
| *Echinochloa spp* | Cockspur or Barnyard grass or Wild millet | Local Tamil name is Kolichchoodan (கோழிச்சூடன்). |  |
| *Cynodon spp* | Scutch grass or Bermuda grass | Local Tamil name is “Padar Pul” (படர்புல்) |  |
| *Digitaria eriantha* | Pangola grass |  |  |
| **Concentrate samples** | | | |
| **Sample** | | **Comments** | |
| Beer waste | | Brewers grains (barley) | |
| Brand1^a^ dairy cow feed | | Commercially available pelleted feed for dairy cows. Made in Sri Lanka | |
| Brand2^a^ milk mash | | Commercially available barley/coconut residue for dairy cows | |
| Dhal Red Lentil mix | | Made from the pods of red lentils | |
| Rice bran | |  | |
| Rice polish | |  | |
| Wheat bran | |  | |
| Various | | Home/locally made mixes using locally available by-products from coconut, rice or cereal production | |

^a^ Brand names have been removed for ethical and legal reasons

**Table S2-** Continuation

| **Nutritional supplements** | |
| --- | --- |
| **Sample** | **Comments** |
| Brand3^a^ | Commercial amino acid-based nutritional supplement |
| Brand4^a^ | Commercial multi-mineral nutritional supplement |
| Calcium powder/limestone | Calcium carbonate (CaCO_3_) supplement |
| Brand5^a^ | Commercial fertility supplement |
| Brand6^a^ | Commercial multi-mineral nutritional supplement |
| Brand7^a^ | Commercial multi-mineral nutritional supplement |
| Brand8^a^ | Commercial multi-mineral nutritional supplement |

^a^ Brand names have been removed for ethical and legal reasons

Table S3. Standard operating conditions for the
Agilent 7800x inductively coupled plasma mass spectrometer.

| **Parameter** | **Operating Condition** |
| --- | --- |
| RF Power | 1.55 kW |
| Carrier gas flow | 0.8 L/min Ar |
| Dilution gas flow | 0.3 L/min Ar |
| Detection mode | Pulse and analog |
| Nebuliser pump rate | 0.1 rps |
| Collision cell gas flow | 4.8 mL/min He |
| Stabilization time | 30 s |
| Sample uptake time (s) | 50 s |
| Rinse time (s) | 120 s |

Table S4. Results of the analysis of certified reference materials for the validation for the determination of metals and metalloids by ICP-MS,
and values of the limits of detection (LOD, instrumental and method)

| **Analyte** | **SRM 1640a** | | **SRM 1643F** | | **LOD** | |
| --- | --- | --- | --- | --- | --- | --- |
|  | **Certified (µg/L)** | **Measured mean ± SD (µg/L, n=3)** | **Certified**  **(µg/L)** | **Measured mean ± SD (µg/L, n=3)** | **Instrument (µg/L)** | **Method**  **(mg kg^-1^)** |
| **Na** | 3.14 ± 0.031 | 2.98 ± 0.22 | 18.64 ± 0.24 | 18.67 ± 0.04 | 49 | 4 |
| **Mg** | 1.06 ± 0.0041 | 1.05 ± 0.01 | 7.38 ± 0.058 | 7.38 ± 0.01 | 5 | 0.3 |
| **K** | 0.58 ± 0.0023 | 0.55 ± 0.01 | 1.91 ± 0.0009 | 1.92 ± 0.03 | 55 | 2.5 |
| **Ca** | 5.62 ± 0.021 | 5.67 ± 0.08 | 29.14 ± 0.32 | 29.13 ± 0.02 | 121 | 5 |
| **Mn** | 40.39 ± 0.36 | 36.1 ± 2.1 | 37.14 ± 0.60 | 34.5 ± 0.6 | 0.06 | 0.01 |
| **Fe** | 36.80 ± 1.8 | 37.3 ± 0.5 | 93.44 ± 0.78 | 83.4 ± 6.3 | 7 | 0.35 |
| **V** | 15.05 ± 0.25 | 15.3 ± 0.3 | 36.07 ± 0.28 | 31.9 ± 2.6 | 0.04 | 0.006 |
| **Cr** | 40.54 ± 0.30 | 36.0 ± 2.4 | 18.50 ± 0.10 | 18.8 ± 0.3 | 0.05 | 0.008 |
| **Co** | 20.24 ± 0.24 | 19.0 ±0.6 | 25.30 ± 0.17 | 22.8 ± 1.1 | 0.04 | 0.007 |
| **Ni** | 25.32 ± 0.14 | 25.8 ± 0.1 | 59.80 ± 1.4 | 52.2 ± 3.0 | 0.06 | 0.01 |
| **Cu** | 85.75 ± 0.51 | 78.8 ± 6.1 | 21.66 ± 0.71 | 22.7 ± 1.2 | 0.07 | 0.01 |
| **Zn** | 55.64 ± 0.35 | 51.7 ± 1.3 | 74.40 ± 1.7 | 67.1 ± 4.2 | 0.1 | 0.02 |
| **Se** | 20.13 ± 0.17 | 13.2 ± 2.5 | 11.7 ± 0.081 | 10.2 ± 1.3 | 0.01 | 0.003 |
| **Mo** | 45.60 ± 0.61 | 44.6 ± 0.3 | 115.30 ± 1.7 | 105 ± 9 | 0.07 | 0.02 |
| **As** | 8.075 ± 0.046 | 8.00 ± 0.2 | 57.42 ± 0.38 | 57.3 ± 0.8 | 0.2 | 0.04 |
| **Cd** | 3.99 ± 0.074 | 3.83 ± 0.03 | 5.89 ± 0.13 | 5.47 ± 0.4 | 0.01 | 0.002 |
| **Pb** | 12.10 ± 0.05 | 11.1 ± 0.9 | 18.49 ± 0.084 | 17.4 ± 1.5 | 0.02 | 0.002 |

**Table S5-** Summary results for all analyte in milk samples

| **Region (number of samples)** | **Parameter** | **Elemental concentrations** | | | | | | | | | | | | | | | | | |
| --- | --- | --- | --- | --- | --- | --- | --- | --- | --- | --- | --- | --- | --- | --- | --- | --- | --- | --- | --- |
|  |  | **Major elements (mg/L)** | | | | **Trace elements (µg/L)** | | | | | | | | | | | | | |
|  |  | **Na** | **Mg** | **K** | **Ca** | **Mn** | **Fe** | **V** | **Cr** | **Co** | **Ni** | **Cu** | **Zn** | **Se** | **Mo** | **I** | **As** | **Cd** | **Pb** |
| Vavuniya (17) | Minimum | 254 | 79.0 | 1267 | 845 | 23.5 | 268 | 0.21 | 0.18 | 0.08 | 1.98 | 34.7 | 3012 | 6.89 | 33.6 | 59.8 | 0.56 | 0.03 | 0.36 |
|  | Maximum | 598 | 128 | 1489 | 12060 | 42.7 | 1201 | 1.45 | 3.34 | 2.67 | 5.20 | 109 | 5423 | 19.0 | 59.7 | 160 | 1.65 | 0.19 | 2.89 |
|  | Median | 391 | 96.0 | 1377 | 967 | 29.5 | 379 | 0.38 | 0.32 | 0.29 | 3.07 | 52.3 | 3987 | 9.67 | 38.9 | 93.6 | 0.87 | 0.06 | 0.47 |
|  | Average | 378 | 98.2 | 1366 | 998 | 31.3 | 609 | 0.66 | 0.84 | 0.57 | 3.18 | 63.1 | 4080 | 11.4 | 41.4 | 104 | 0.94 | 0.07 | 0.87 |
|  | SD | 104 | 13.6 | 71 | 132 | 5.8 | 386 | 0.45 | 0.92 | 0.64 | 0.98 | 25.1 | 765 | 4.03 | 7.11 | 31 | 0.29 | 0.04 | 0.73 |
| Jaffna (19) | Minimum | 298 | 71.0 | 1183 | 756 | 23.7 | 216 | 0.23 | 0.18 | 0.08 | 2.19 | 32.8 | 3189 | 6.34 | 33.5 | 65.8 | 0.56 | 0.05 | 0.34 |
|  | Maximum | 477 | 113 | 1456 | 1128 | 38.5 | 1078 | 1.03 | 1.45 | 1.56 | 5.78 | 78.8 | 4987 | 14.5 | 49.8 | 123 | 1.78 | 0.14 | 1.34 |
|  | Median | 387 | 84.0 | 1328 | 866 | 32.6 | 328 | 0.34 | 0.43 | 0.38 | 3.66 | 40.7 | 3652 | 8.34 | 43.7 | 81.4 | 0.87 | 0.08 | 0.58 |
|  | Average | 381 | 88.0 | 1322 | 891 | 31.7 | 427 | 0.43 | 0.50 | 0.50 | 3.41 | 46.4 | 3765 | 9.21 | 42.1 | 84.9 | 0.91 | 0.09 | 0.66 |
|  | SD | 46 | 12.4 | 85.0 | 103 | 4.5 | 281 | 0.23 | 0.33 | 0.41 | 0.96 | 13.8 | 58 | 2.45 | 5.1 | 15.7 | 0.32 | 0.03 | 0.24 |
| Mannar (20) | Minimum | 308 | 3.00 | 1189 | 713 | 20.4 | 276 | 0.29 | 0.14 | 0.11 | 1.73 | 29.3 | 2548 | 8.67 | 0.72 | 44.5 | 0.54 | 0.05 | 0.19 |
|  | Maximum | 409 | 118 | 1509 | 1034 | 52.9 | 512 | 0.59 | 0.67 | 0.37 | 4.01 | 61.2 | 4120 | 13.2 | 67.3 | 120 | 1.56 | 0.15 | 0.83 |
|  | Median | 340 | 84.0 | 1412 | 864 | 39.2 | 360 | 0.38 | 0.38 | 0.29 | 2.62 | 43.8 | 3659 | 10.0 | 36.0 | 86.8 | 0.80 | 0.08 | 0.35 |
|  | Average | 348 | 84.2 | 1401 | 865 | 39.5 | 365 | 0.40 | 0.42 | 0.28 | 2.58 | 43.1 | 3519 | 10.0 | 36.0 | 84.2 | 0.84 | 0.08 | 0.38 |
|  | SD | 33 | 23.0 | 97 | 88.6 | 8.86 | 68.4 | 0.07 | 0.13 | 0.07 | 0.55 | 7.6 | 430 | 1.1 | 12.3 | 14.7 | 0.26 | 0.02 | 0.15 |
| Kurunegala (16) | Minimum | 317 | 78.0 | 1398 | 783 | 26.5 | 276 | 0.32 | 0.25 | 0.13 | 2.32 | 39.2 | 2578 | 8.75 | 40.2 | 61.3 | 0.34 | 0.08 | 0.39 |
|  | Maximum | 487 | 176 | 1920 | 1289 | 66.8 | 509 | 0.77 | 0.73 | 0.84 | 3.67 | 51.3 | 5120 | 22.1 | 113 | 118 | 1.78 | 0.22 | 1.20 |
|  | Median | 387 | 116 | 1583 | 923 | 43.6 | 386 | 0.45 | 0.44 | 0.35 | 2.66 | 46.8 | 3852 | 15.0 | 71.3 | 87.3 | 0.89 | 0.13 | 0.53 |
|  | Average | 393 | 119 | 1617 | 975 | 42.5 | 383 | 0.47 | 0.44 | 0.40 | 2.75 | 45.7 | 3826 | 14.8 | 73.9 | 89.2 | 0.93 | 0.14 | 0.61 |
|  | SD | 56 | 28 | 156 | 168 | 11.1 | 62 | 0.11 | 0.14 | 0.21 | 0.42 | 4.3 | 746 | 4.6 | 23.3 | 17.0 | 0.39 | 0.04 | 0.21 |
| UK control site (6) | Minimum | 287 | 27.8 | 156 | 168 | 11.1 | 62.5 | 0.11 | 0.14 | 0.08 | 0.42 | 4.28 | 746 | 4.55 | 23.3 | 17.0 | 0.34 | 0.03 | 0.21 |
|  | Maximum | 478 | 176 | 1920 | 1289 | 66.8 | 904 | 0.77 | 0.78 | 0.84 | 4.03 | 81.2 | 5120 | 22.1 | 113 | 118 | 1.78 | 0.22 | 1.20 |
|  | Median | 348 | 98.0 | 1398 | 937 | 37.3 | 383 | 0.43 | 0.43 | 0.35 | 2.75 | 50.2 | 3829 | 11.3 | 56.9 | 89.2 | 0.93 | 0.12 | 0.60 |
|  | Average | 366 | 102 | 1373 | 915 | 35.1 | 418 | 0.40 | 0.42 | 0.37 | 2.70 | 50.5 | 3607 | 12.0 | 63.8 | 87.2 | 1.02 | 0.12 | 0.60 |
|  | SD | 81.1 | 33.4 | 408 | 263 | 13.2 | 216 | 0.19 | 0.20 | 0.23 | 1.03 | 19.4 | 1137 | 4.74 | 28.5 | 28.3 | 0.45 | 0.07 | 0.25 |

**Table S6-** Summary results for all analyte in blood serum samples

| **Region (number of samples)** | **Parameter** | **Elemental concentrations** | | | | | | | | | | | | | | | | | |
| --- | --- | --- | --- | --- | --- | --- | --- | --- | --- | --- | --- | --- | --- | --- | --- | --- | --- | --- | --- |
|  |  | **Major elements (mg/L)** | | | | **Trace elements (µg/L)** | | | | | | | | | | | | | |
|  |  | **Na** | **Mg** | **K** | **Ca** | **Mn** | **Fe** | **V** | **Cr** | **Co** | **Ni** | **Cu** | **Zn** | **Se** | **Mo** | **I** | **As** | **Cd** | **Pb** |
| Vavuniya (30) | Minimum | 3076 | 19.3 | 154 | 104 | 0.23 | 890 | 0.13 | 0.08 | 0.06 | 0.93 | 698 | 756 | 155 | 0.52 | 18.6 | 0.98 | 0.05 | 3.20 |
|  | Maximum | 3487 | 24.3 | 198 | 132 | 1.09 | 1278 | 0.65 | 0.72 | 0.31 | 3.08 | 1376 | 1288 | 609 | 1.78 | 66.8 | 3.76 | 0.55 | 11.90 |
|  | Median | 3278 | 22.3 | 180 | 118 | 0.45 | 1039 | 0.23 | 0.24 | 0.19 | 1.90 | 894 | 895 | 275 | 0.83 | 43.2 | 1.56 | 0.15 | 4.20 |
|  | Average | 3253 | 22.1 | 178 | 117 | 0.49 | 1054 | 0.29 | 0.28 | 0.19 | 1.89 | 943 | 914 | 304 | 0.85 | 45.2 | 1.76 | 0.19 | 4.98 |
|  | SD | 112 | 1.4 | 12 | 7 | 0.20 | 105 | 0.15 | 0.17 | 0.06 | 0.57 | 164 | 136 | 110 | 0.22 | 9.8 | 0.75 | 0.14 | 2.09 |
| Jaffna (30) | Minimum | 3065 | 20.6 | 166 | 107 | 0.32 | 874 | 0.09 | 0.12 | 0.09 | 1.08 | 779 | 765 | 178 | 0.98 | 32.7 | 0.78 | 0.09 | 2.70 |
|  | Maximum | 3866 | 34.8 | 206 | 138 | 0.76 | 1213 | 0.56 | 0.65 | 0.65 | 2.78 | 1044 | 1208 | 563 | 3.03 | 66.6 | 3.19 | 0.54 | 8.30 |
|  | Median | 3285 | 23.6 | 184 | 118 | 0.52 | 1006 | 0.23 | 0.25 | 0.22 | 1.56 | 874 | 945 | 309 | 1.45 | 43.8 | 1.48 | 0.23 | 4.40 |
|  | Average | 3351 | 24.0 | 185 | 121 | 0.53 | 1015 | 0.25 | 0.28 | 0.27 | 1.65 | 888 | 957 | 312 | 1.71 | 45.0 | 1.61 | 0.26 | 4.76 |
|  | SD | 227 | 3.2 | 9 | 9 | 0.12 | 86 | 0.10 | 0.12 | 0.12 | 0.40 | 79 | 109 | 102 | 0.56 | 9.3 | 0.60 | 0.09 | 1.46 |
| Mannar (30) | Minimum | 3008 | 17.4 | 160 | 102 | 0.21 | 703 | 0.11 | 0.16 | 0.08 | 0.97 | 665 | 657 | 134 | 0.33 | 32.8 | 0.93 | 0.08 | 1.40 |
|  | Maximum | 3426 | 29.7 | 196 | 132 | 0.76 | 1129 | 0.54 | 0.88 | 0.56 | 4.50 | 1245 | 1654 | 344 | 1.45 | 78.5 | 2.78 | 0.43 | 8.90 |
|  | Median | 3108 | 21.6 | 178 | 113 | 0.33 | 907 | 0.28 | 0.38 | 0.23 | 1.56 | 785 | 850 | 214 | 0.46 | 40.8 | 1.45 | 0.14 | 3.55 |
|  | Average | 3157 | 21.6 | 178 | 114 | 0.39 | 910 | 0.29 | 0.39 | 0.23 | 1.68 | 842 | 883 | 217 | 0.53 | 44.0 | 1.58 | 0.17 | 4.02 |
|  | SD | 121 | 2.3 | 9 | 7 | 0.15 | 104 | 0.10 | 0.16 | 0.10 | 0.81 | 167 | 201 | 43 | 0.22 | 11.2 | 0.53 | 0.08 | 2.05 |
| Kurunegala (30) | Minimum | 3021 | 17.9 | 152 | 104 | 0.23 | 812 | 0.12 | 0.09 | 0.06 | 0.89 | 563 | 657 | 132 | 0.52 | 30.2 | 0.87 | 0.07 | 1.40 |
|  | Maximum | 3534 | 26.3 | 196 | 135 | 0.78 | 1228 | 0.53 | 0.48 | 0.56 | 4.34 | 1321 | 1287 | 321 | 1.65 | 72.1 | 3.24 | 0.31 | 9.40 |
|  | Median | 3207 | 21.6 | 176 | 117 | 0.50 | 988 | 0.22 | 0.28 | 0.21 | 1.56 | 874 | 913 | 200 | 0.72 | 43.9 | 1.63 | 0.16 | 2.90 |
|  | Average | 3198 | 21.9 | 173 | 116 | 0.49 | 992 | 0.24 | 0.28 | 0.21 | 1.69 | 878 | 916 | 212 | 0.81 | 45.7 | 1.73 | 0.17 | 3.69 |
|  | SD | 103 | 1.9 | 10 | 7 | 0.13 | 96 | 0.10 | 0.10 | 0.09 | 0.78 | 173 | 138 | 37 | 0.30 | 10.2 | 0.56 | 0.06 | 2.06 |
| UK control site (6) | Minimum | 3008 | 20.7 | 158 | 103 | 0.38 | 938 | 0.17 | 0.07 | 0.06 | 1.60 | 663 | 729 | 158 | 0.57 | 38.9 | 1.03 | 0.05 | 2.10 |
|  | Maximum | 3876 | 23.8 | 193 | 128 | 0.78 | 1183 | 0.37 | 0.67 | 0.32 | 3.20 | 1078 | 1100 | 330 | 0.98 | 53.5 | 3.02 | 0.19 | 5.40 |
|  | Median | 3232 | 21.8 | 169 | 117 | 0.60 | 1042 | 0.24 | 0.17 | 0.17 | 2.50 | 908 | 885 | 215 | 0.89 | 44.8 | 1.63 | 0.09 | 4.05 |
|  | Average | 3282 | 22.1 | 173 | 117 | 0.59 | 1051 | 0.26 | 0.26 | 0.19 | 2.44 | 909 | 912 | 241 | 0.82 | 45.8 | 1.93 | 0.11 | 3.92 |
|  | SD | 319 | 1.53 | 14.5 | 9 | 0.16 | 98 | 0.09 | 0.22 | 0.10 | 0.67 | 144 | 155 | 68.2 | 0.16 | 6.2 | 0.79 | 0.06 | 1.20 |

**Table S7-** Summary results for all analytes in EDTA-whole blood samples

| **Region (number of samples)** | **Parameter** | **Elemental concentrations** | | | | | | | | | | | | | | | |
| --- | --- | --- | --- | --- | --- | --- | --- | --- | --- | --- | --- | --- | --- | --- | --- | --- | --- |
|  |  | **Major elements (mg/L)** | | **Trace elements  (µg/L)** | | | | | | | | | | | | | |
|  |  | **Mg** | **Ca** | **Mn** | **Fe** | **V** | **Cr** | **Co** | **Ni** | **Cu** | **Zn** | **Se** | **Mo** | **I** | **As** | **Cd** | **Pb** |
| Vavuniya (30) | Minimum | 4.60 | 47.3 | 3.10 | 389 | 0.14 | 0.09 | 0.06 | 0.19 | 830 | 5460 | 78.0 | 0.45 | 32.4 | 10.3 | 0.16 | 3.70 |
|  | Maximum | 52.1 | 78.3 | 6.30 | 613 | 0.51 | 0.45 | 0.23 | 2.28 | 2780 | 10450 | 123 | 1.56 | 78.3 | 44.3 | 0.73 | 20.20 |
|  | Median | 33.6 | 63.8 | 4.20 | 512 | 0.20 | 0.18 | 0.16 | 1.45 | 1000 | 7165 | 93.0 | 0.82 | 48.4 | 16.0 | 0.30 | 6.80 |
|  | Average | 35.5 | 62.3 | 4.32 | 507 | 0.24 | 0.22 | 0.15 | 1.44 | 1351 | 7298 | 95.4 | 0.83 | 50.6 | 18.3 | 0.34 | 7.97 |
|  | SD | 10.0 | 10.6 | 0.92 | 51 | 0.09 | 0.09 | 0.04 | 0.43 | 582 | 1071 | 12.0 | 0.23 | 11.7 | 7.9 | 0.16 | 4.05 |
| Jaffna (30) | Minimum | 24.5 | 45.6 | 2.30 | 435. | 0.11 | 0.09 | 0.11 | 0.67 | 850 | 6050 | 81.0 | 0.83 | 33.6 | 9.30 | 0.18 | 3.60 |
|  | Maximum | 56.3 | 80.7 | 5.60 | 1213 | 0.34 | 0.45 | 0.55 | 1.87 | 1770 | 10020 | 123 | 2.23 | 89.3 | 28.4 | 0.72 | 14.50 |
|  | Median | 39.5 | 67.5 | 4.30 | 498 | 0.18 | 0.20 | 0.18 | 1.32 | 950 | 7230 | 94.0 | 1.32 | 47.4 | 15.4 | 0.35 | 7.50 |
|  | Average | 40.8 | 65.9 | 4.30 | 525 | 0.20 | 0.23 | 0.21 | 1.31 | 1091 | 7457 | 95.1 | 1.44 | 50.3 | 17.5 | 0.38 | 7.69 |
|  | SD | 9.5 | 9.9 | 0.90 | 139 | 0.06 | 0.09 | 0.10 | 0.28 | 260 | 873 | 11.1 | 0.42 | 11.3 | 5.8 | 0.11 | 2.88 |
| Mannar (30) | Minimum | 38.5 | 43.7 | 1.80 | 378 | 0.13 | 0.15 | 0.09 | 0.67 | 720 | 5100 | 78.0 | 0.28 | 32.4 | 9.4 | 0.16 | 0.17 |
|  | Maximum | 55.6 | 67.8 | 5.60 | 515 | 0.45 | 0.66 | 0.44 | 2.88 | 2430 | 10980 | 101 | 1.09 | 98.3 | 36.4 | 0.44 | 15.40 |
|  | Median | 43.6 | 56.8 | 2.80 | 450 | 0.23 | 0.32 | 0.18 | 1.28 | 850 | 7130 | 85.0 | 0.44 | 45.3 | 16.5 | 0.28 | 5.30 |
|  | Average | 44.0 | 56.3 | 3.09 | 451 | 0.24 | 0.31 | 0.19 | 1.29 | 1058 | 7191 | 86.3 | 0.50 | 48.4 | 18.6 | 0.28 | 6.25 |
|  | SD | 3. 9 | 6.2 | 0.95 | 37 | 0.09 | 0.11 | 0.07 | 0.49 | 427 | 1221 | 4.8 | 0.17 | 14.0 | 6.1 | 0.08 | 3.85 |
| Kurunegala (30) | Minimum | 24.5 | 48.5 | 2.20 | 4120 | 0.12 | 0.15 | 0.09 | 0.73 | 670 | 5340 | 76.0 | 0.42 | 30.8 | 12.4 | 0.15 | 2.80 |
|  | Maximum | 53.2 | 73.6 | 6.20 | 612 | 0.43 | 0.37 | 0.34 | 3.29 | 2090 | 9890 | 97.0 | 1.38 | 77.5 | 33.0 | 0.55 | 17.50 |
|  | Median | 39.8 | 56.0 | 4.10 | 479 | 0.18 | 0.24 | 0.16 | 1.29 | 940 | 7450 | 84.0 | 0.66 | 45.7 | 18.9 | 0.29 | 4.80 |
|  | Average | 40.5 | 58.1 | 4.11 | 486 | 0.20 | 0.25 | 0.16 | 1.38 | 1088 | 7379 | 85.1 | 0.71 | 49.1 | 19.5 | 0.28 | 6.34 |
|  | SD | 6.8 | 6.8 | 1.10 | 44 | 0.08 | 0.06 | 0.05 | 0.53 | 386 | 990 | 4.5 | 0.21 | 12.5 | 5.0 | 0.09 | 4.17 |
| UK control site (6) | Minimum | 18.0 | 55.8 | 1.90 | 385 | 0.10 | 0.18 | 0.13 | 1.20 | 720 | 7410 | 61.0 | 0.53 | 34.2 | 11.9 | 0.06 | 1.20 |
|  | Maximum | 36.3 | 76.4 | 4.90 | 585 | 0.31 | 0.73 | 0.32 | 3.20 | 1860 | 9890 | 97.0 | 0.93 | 57.2 | 27.8 | 0.28 | 5.20 |
|  | Median | 21.8 | 60.2 | 3.00 | 492 | 0.18 | 0.32 | 0.20 | 2.27 | 1130 | 9045 | 77.5 | 0.74 | 39.6 | 15.1 | 0.13 | 3.30 |
|  | Average | 23.9 | 63.9 | 3.10 | 482 | 0.20 | 0.41 | 0.21 | 2.20 | 1173 | 8832 | 77.7 | 0.75 | 43.4 | 18.1 | 0.14 | 3.40 |
|  | SD | 7.3 | 9.0 | 1.07 | 71 | 0.07 | 0.23 | 0.07 | 0.78 | 402 | 952 | 14.8 | 0.15 | 8.83 | 6.9 | 0.08 | 1.47 |

**Table S8-** Summary results for all analytes in hair samples

| **Region (number of samples)** | **Parameter** | **Elemental concentrations** | | | | | | | | | | | | | | | | | |
| --- | --- | --- | --- | --- | --- | --- | --- | --- | --- | --- | --- | --- | --- | --- | --- | --- | --- | --- | --- |
|  |  | **Major elements (mg/kg d.w)** | | | | **Trace elements (mg/kg d.w)** | | | | | | | | | | | | | |
|  |  | **Na** | **Mg** | **K** | **Ca** | **Mn** | **Fe** | **V** | **Cr** | **Co** | **Ni** | **Cu** | **Zn** | **Se** | **Mo** | **I** | **As** | **Cd** | **Pb** |
| Vavuniya (30) | Minimum | 77.8 | 11.2 | 48.3 | 90.4 | 0.11 | 13.4 | 0.07 | 0.09 | 0.12 | 0.78 | 7.30 | 98.6 | 0.34 | 0.21 | 0.21 | 0.08 | 0.01 | 0.18 |
|  | Maximum | 238 | 45.2 | 110 | 619 | 0.76 | 78.3 | 0.67 | 1.45 | 0.51 | 4.54 | 22.4 | 223 | 1.77 | 0.60 | 0.63 | 0.38 | 0.16 | 3.80 |
|  | Median | 163 | 17.4 | 80.9 | 356 | 0.28 | 26.6 | 0.20 | 0.43 | 0.33 | 1.62 | 13.4 | 166 | 0.64 | 0.39 | 0.40 | 0.17 | 0.04 | 0.41 |
|  | Average | 152 | 19.4 | 82.7 | 351 | 0.31 | 29.0 | 0.23 | 0.51 | 0.32 | 1.91 | 14.0 | 164 | 0.78 | 0.39 | 0.41 | 0.19 | 0.05 | 0.74 |
|  | SD | 44 | 7.2 | 15.3 | 148 | 0.19 | 15.6 | 0.14 | 0.33 | 0.10 | 0.94 | 3.7 | 29.4 | 0.40 | 0.08 | 0.10 | 0.09 | 0.03 | 0.93 |
| Jaffna (30) | Minimum | 60.4 | 12.6 | 45.6 | 65.3 | 0.15 | 13.5 | 0.08 | 0.09 | 0.12 | 0.87 | 7.60 | 98.3 | 0.34 | 0.21 | 0.33 | 0.07 | 0.01 | 0.15 |
|  | Maximum | 309 | 46.4 | 144 | 893 | 0.89 | 76.3 | 0.53 | 2.13 | 1.21 | 3.23 | 22.3 | 429 | 2.45 | 0.78 | 0.67 | 0.36 | 0.07 | 1.55 |
|  | Median | 149 | 20.4 | 93.4 | 422 | 0.32 | 24.0 | 0.18 | 0.44 | 0.45 | 1.33 | 13.0 | 180 | 0.89 | 0.39 | 0.47 | 0.15 | 0.04 | 0.31 |
|  | Average | 158 | 23.1 | 93.8 | 430 | 0.34 | 29.8 | 0.20 | 0.61 | 0.54 | 1.42 | 13.1 | 184 | 0.95 | 0.45 | 0.46 | 0.17 | 0.03 | 0.44 |
|  | SD | 79 | 8.5 | 19.5 | 213 | 0.16 | 16.4 | 0.11 | 0.49 | 0.26 | 0.48 | 3.4 | 54 | 0.51 | 0.15 | 0.10 | 0.08 | 0.02 | 0.34 |
| Mannar (30) | Minimum | 32.4 | 11.2 | 56.3 | 65.8 | 0.08 | 11.2 | 0.08 | 0.14 | 0.12 | 0.67 | 7.30 | 78.3 | 0.39 | 0.16 | 0.28 | 0.08 | 0.00 | 0.12 |
|  | Maximum | 893 | 35.7 | 106 | 589 | 0.46 | 34.5 | 0.45 | 2.33 | 2.12 | 2.33 | 26.5 | 422 | 1.77 | 0.56 | 0.72 | 0.32 | 0.06 | 3.67 |
|  | Median | 84.1 | 18.6 | 77.8 | 231 | 0.18 | 19.8 | 0.22 | 0.67 | 0.44 | 1.20 | 9.40 | 155 | 0.66 | 0.25 | 0.44 | 0.14 | 0.01 | 0.28 |
|  | Average | 125 | 19.6 | 79.1 | 255 | 0.22 | 19.9 | 0.24 | 0.80 | 0.48 | 1.23 | 11.6 | 160 | 0.65 | 0.28 | 0.46 | 0.14 | 0.02 | 0.60 |
|  | SD | 155 | 5.4 | 14.3 | 140 | 0.10 | 5.5 | 0.10 | 0.52 | 0.36 | 0.36 | 4.7 | 74 | 0.25 | 0.11 | 0.09 | 0.05 | 0.01 | 0.91 |
| Kurunegala (30) | Minimum | 65.0 | 12.4 | 48.5 | 79.3 | 0.09 | 13.4 | 0.07 | 0.09 | 0.08 | 0.73 | 6.50 | 102 | 0.33 | 0.23 | 0.28 | 0.06 | 0.01 | 0.09 |
|  | Maximum | 309 | 34.6 | 144 | 628 | 0.78 | 45.6 | 0.65 | 1.20 | 2.19 | 3.33 | 19.3 | 950 | 1.66 | 0.73 | 0.66 | 0.35 | 0.06 | 2.89 |
|  | Median | 132 | 18.9 | 70.4 | 243 | 0.28 | 21.4 | 0.19 | 0.43 | 0.38 | 1.45 | 10.1 | 170 | 0.60 | 0.39 | 0.48 | 0.14 | 0.02 | 0.21 |
|  | Average | 140 | 20.3 | 75.9 | 294 | 0.29 | 24.1 | 0.22 | 0.46 | 0.42 | 1.58 | 11.0 | 194 | 0.64 | 0.42 | 0.47 | 0.15 | 0.02 | 0.42 |
|  | SD | 57 | 5.3 | 18.9 | 154 | 0.13 | 8.4 | 0.13 | 0.24 | 0.37 | 0.71 | 3.0 | 146 | 0.23 | 0.12 | 0.09 | 0.07 | 0.02 | 0.61 |
| UK control site (6) | Minimum | 86.2 | 18.3 | 65.3 | 167 | 0.20 | 18.4 | 0.15 | 0.20 | 0.16 | 1.78 | 8.80 | 112 | 0.29 | 0.73 | 0.30 | 0.17 | 0.02 | 0.03 |
|  | Maximum | 198 | 38.7 | 104 | 423 | 0.73 | 39.7 | 0.45 | 0.67 | 0.38 | 4.56 | 20.3 | 183 | 0.89 | 0.95 | 0.49 | 0.34 | 0.05 | 0.10 |
|  | Median | 177 | 23.8 | 79.5 | 333 | 0.35 | 27.6 | 0.28 | 0.32 | 0.23 | 2.75 | 11.2 | 152 | 0.41 | 0.86 | 0.38 | 0.29 | 0.04 | 0.09 |
|  | Average | 155 | 26.0 | 82.4 | 310 | 0.39 | 27.8 | 0.27 | 0.38 | 0.24 | 3.00 | 13.3 | 153 | 0.51 | 0.84 | 0.39 | 0.27 | 0.04 | 0.07 |
|  | SD | 49 | 8.5 | 15.9 | 95.3 | 0.19 | 8.9 | 0.11 | 0.19 | 0.08 | 1.23 | 4.4 | 27 | 0.23 | 0.09 | 0.07 | 0.07 | 0.01 | 0.03 |

**Table S9-** Summary results for all analytes in the local fodder used by local dairy farmers in the sampling area,
(Vavuniya, Mannar, Jaffna and Kurunegala, in Sri Lanka).

| **Feed (number of samples)** | **Parameter** | **Elemental concentrations** | | | | | | | | | | | | | | | |
| --- | --- | --- | --- | --- | --- | --- | --- | --- | --- | --- | --- | --- | --- | --- | --- | --- | --- |
|  |  | **Major elements (g/kg d.w)** | | | | **Trace elements (mg/kg d.w)** | | | | | | | | | | | |
|  |  | **Na** | **Mg** | **K** | **Ca** | **V** | **Cr** | **Mn** | **Fe** | **Co** | **Ni** | **Cu** | **Zn** | **Se** | **Mo** | **Cd** | **Pb** |
| Azola (6) | Minimum | 12.0 | 4.13 | 6.05 | 15.0 | 1.84 | 1.57 | 567 | 546 | 1.17 | 1.47 | 3.81 | 24.4 | 0.42 | <LOD | <LOD | 0.37 |
|  | Maximum | 79.1 | 13.5 | 51.3 | 157 | 12.6 | 6.62 | 2317 | 2462 | 13.4 | 9.07 | 15.6 | 51.6 | 1.72 | 1.68 | 0.24 | 2.48 |
|  | Median | 15.7 | 10.9 | 23.1 | 41.5 | 2.64 | 2.75 | 927 | 896 | 1.55 | 2.43 | 6.29 | 33.4 | 0.59 | - | - | 1.56 |
|  | Average | 29.9 | 9.48 | 27.5 | 60.0 | 5.16 | 3.39 | 1268 | 1132 | 4.06 | 3.92 | 7.41 | 35.0 | 0.84 | - | - | 1.48 |
|  | SD | 27.0 | 3.55 | 17.0 | 54.4 | 4.50 | 1.93 | 840 | 732 | 4.85 | 3.04 | 4.49 | 9.8 | 0.54 | - | - | 0.79 |
| CO3 (35) | Minimum | 0.11 | 1.50 | 18.2 | 2.26 | <LOD | 0.77 | 14.5 | 47.6 | <LOD | <LOD | 2.13 | 11.9 | 0.30 | <LOD | <LOD | <LOD |
|  | Maximum | 15.4 | 11.0 | 102 | 64.8 | 3.70 | 2.85 | 1663 | 743 | 5.06 | 6.66 | 43.8 | 165 | 1.16 | 4.96 | <LOD | 1.51 |
|  | Median | 0.64 | 2.58 | 44.3 | 6.26 | - | 1.38 | 55.2 | 170 | - | - | 8.47 | 28.1 | 0.57 | - | - | - |
|  | Average | 1.37 | 3.18 | 49.1 | 8.96 | - | 1.41 | 112 | 232 | - | - | 9.58 | 36.6 | 0.59 | - | - | - |
|  | SD | 2.64 | 1.75 | 21.0 | 11.29 | - | 0.51 | 271 | 157 | - | - | 7.58 | 28.9 | 0.15 | - | - | - |
| Glicidia (23) | Minimum | 0.13 | 2.40 | 6.19 | 2.96 | <LOD | 0.85 | 26.5 | 127 | <LOD | 1.09 | 2.41 | 12.2 | <LOD | <LOD | <LOD | <LOD |
|  | Maximum | 55.6 | 14.0 | 122 | 65.0 | 10.8 | 3.19 | 501 | 1432 | - | 6.10 | 202 | 1475 | 9.74 | 1.57 | 1.14 | 3.02 |
|  | Median | 0.58 | 6.63 | 22.5 | 23.8 | - | 1.36 | 66.4 | 280 | - | 1.73 | 4.80 | 18.6 | - | - | - | - |
|  | Average | 4.0 | 7.11 | 31.6 | 26.6 | - | 1.54 | 104 | 403 | - | 2.10 | 14.2 | 86.0 | - | - | - | - |
|  | SD | 11.4 | 3.37 | 24.8 | 17.2 | - | 0.65 | 112 | 315 | - | 1.15 | 41.0 | 303 | - | - | - | - |
| Ipil-Ipil (13) | Minimum | 2.75 | 9.04 | 6.98 | 0.78 | 1.13 | 38.7 | 217 | <LOD | 1.26 | 5.08 | 14.5 | 0.48 | <LOD | <LOD | 0.37 | 2.75 |
|  | Maximum | 8.27 | 74.2 | 53.7 | 3.16 | 3.64 | 139 | 1238 | 1.36 | 5.09 | 50.7 | 107 | 1.13 | 2.28 | <LOD | 1.73 | 8.27 |
|  | Median | 4.42 | 23.2 | 29.9 | 1.29 | 1.73 | 74.4 | 383 | - | 1.86 | 9.47 | 19.9 | 0.64 | - |  | 0.40 | 4.42 |
|  | Average | 4.96 | 31.7 | 31.0 | 1.48 | 1.89 | 81.4 | 489 | - | 2.49 | 13.5 | 35.8 | 0.67 | - |  | 0.57 | 4.96 |
|  | SD | 1.90 | 19.0 | 14.5 | 0.73 | 0.68 | 29.0 | 300 | - | 1.26 | 13.0 | 30.5 | 0.17 | - |  | 0.38 | 1.90 |
| Korai (15) | Minimum | 2.70 | 1.54 | 21.8 | 4.87 | 0.79 | 1.18 | 30.0 | 234 | <LOD | 1.67 | 3.86 | 18.40 | <LOD | <LOD | <LOD | <LOD |
|  | Maximum | 32.6 | 7.94 | 94.5 | 43.5 | 7.43 | 7.69 | 168 | 3262 | 1.66 | 5.67 | 14.0 | 56.9 | 1.89 | 2.90 | 1.47 | 1.33 |
|  | Median | 8.59 | 3.83 | 34.2 | 8.02 | 1.51 | 2.33 | 79.5 | 591 | - | 2.15 | 8.64 | 26.6 | - | - | - | - |
|  | Average | 9.57 | 4.05 | 37.3 | 11.6 | 2.82 | 3.22 | 85.4 | 1214 | - | 2.94 | 8.68 | 30.9 | - | - | - | - |
|  | SD | 7.09 | 1.70 | 17.1 | 11.1 | 2.17 | 2.21 | 34.6 | 1086 | - | 1.41 | 2.94 | 11.8 | - | - | - | - |
| Sarana (12) | Minimum | 2.21 | 1.92 | 12.9 | 2.09 | 0.65 | 1.22 | 45.9 | 101 | <LOD | 1.44 | 3.74 | 19.2 | <LOD | <LOD | <LOD | 0.19 |
|  | Maximum | 38.1 | 9.53 | 63.5 | 58.3 | 6.10 | 3.79 | 1158 | 1440 | 5.19 | 4.37 | 15.2 | 65.6 | 0.96 | 2.39 | 1.31 | 0.81 |
|  | Median | 12.4 | 6.83 | 41.9 | 9.78 | 1.65 | 1.95 | 90.1 | 526 | - | 2.23 | 6.40 | 29.0 | - | - | - | 0.33 |
|  | Average | 16.1 | 6.02 | 40.5 | 14.4 | 2.25 | 2.25 | 223 | 679 | - | 2.70 | 7.07 | 31.1 | - | - | - | 0.42 |
|  | SD | 12.7 | 2.71 | 16.7 | 15.4 | 1.60 | 0.89 | 321 | 441 | - | 1.05 | 3.24 | 12.4 | - | - | - | 0.22 |
| Paddy straw (27) | Minimum | 0.17 | 0.72 | 6.60 | 0.81 | <LOD | <LOD | 30.9 | 41.7 | <LOD | <LOD | <LOD | 12.6 | <LOD | <LOD | <LOD | <LOD |
|  | Maximum | 25.3 | 7.29 | 46.6 | 50.3 | 5.92 | 38.98 | 926 | 987 | 5.26 | 4.38 | 14.49 | 108 | 1.14 | 4.06 | 0.49 | 5.72 |
|  | Median | 1.52 | 1.79 | 19.6 | 2.67 | - | - | 195 | 123 | - | - | - | 27.5 | - | - | - | - |
|  | Average | 3.71 | 2.19 | 21.4 | 5.01 | - | - | 244 | 216 | - | - | - | 36.6 | - | - | - | - |
|  | SD | 5.92 | 1.50 | 10.39 | 9.39 | - | - | 189 | 215 | - | - | - | 23.9 | - | - | - | - |

**Table S9-** Continuation.

| **Feed (number of samples)** | **Parameter** | **Elemental concentrations** | | | | | | | | | | | | | | | |
| --- | --- | --- | --- | --- | --- | --- | --- | --- | --- | --- | --- | --- | --- | --- | --- | --- | --- |
|  |  | **Major elements (g/kg d.w)** | | | | **Trace elements (mg/kg d.w)** | | | | | | | | | | | |
|  |  | **Na** | **Mg** | **K** | **Ca** | **V** | **Cr** | **Mn** | **Fe** | **Co** | **Ni** | **Cu** | **Zn** | **Se** | **Mo** | **Cd** | **Pb** |
| Kolicchoodan (10) | Minimum | 0.95 | 2.48 | 20.5 | 4.38 | <LOD | 0.78 | 39.6 | 95.3 | <LOD | 0.91 | 3.49 | 19.8 | <LOD | <LOD | <LOD | 0.21 |
|  | Maximum | 46.2 | 19.9 | 82.2 | 33.2 | 3.91 | 3.53 | 536 | 1370 | 1.34 | 3.69 | 15.1 | 62.1 | 0.76 | 2.06 | 0.23 | 18.08 |
|  | Median | 9.06 | 6.07 | 36.7 | 10.7 | - | 1.96 | 122 | 518 | - | 2.37 | 7.00 | 29.3 | - | - | - | 0.39 |
|  | Average | 16.8 | 7.51 | 42.4 | 14.2 | - | 1.96 | 220 | 578 | - | 2.32 | 7.51 | 31.7 | - | - | - | 2.20 |
|  | SD | 16.8 | 5.22 | 21.0 | 10.6 | - | 0.89 | 194 | 379 | - | 0.75 | 3.10 | 11.9 | - | - | - | 5.58 |
| Padarpul (5) | Minimum | 1.91 | 3.13 | 21.4 | 3.18 | 0.69 | 1.19 | 43.0 | 115 | 0.82 | 1.21 | 4.27 | 13.9 | <LOD | <LOD | <LOD | <LOD |
|  | Maximum | 60.6 | 15.6 | 42.5 | 40.7 | 2.60 | 2.31 | 477 | 823 | 1.62 | 3.32 | 7.68 | 41.5 | 1.00 | 2.78 | 0.49 | 0.50 |
|  | Median | 9.48 | 5.02 | 40.0 | 6.31 | 1.61 | 1.93 | 80.6 | 471 | 0.95 | 2.27 | 5.91 | 20.2 | - | - | - | - |
|  | Average | 17.5 | 7.44 | 35.6 | 13.7 | 1.59 | 1.87 | 163 | 466 | 1.07 | 2.41 | 6.02 | 23.5 | - | - | - | - |
|  | SD | 24.3 | 5.16 | 8.48 | 15.5 | 0.68 | 0.42 | 180 | 256 | 0.32 | 0.88 | 1.36 | 10.7 | - | - | - | - |
| Local grass -Vavuniya (4) | Minimum | 1.07 | 3.67 | 13.4 | 6.92 | 1.00 | 1.21 | 44.7 | 256 | <LOD | 1.43 | 5.29 | 16.5 | 0.39 | <LOD | <LOD | 0.40 |
|  | Maximum | 3.43 | 4.55 | 96.6 | 28.1 | 3.58 | 2.64 | 95.3 | 1589 | 1.07 | 5.35 | 16.0 | 55.4 | 0.49 | 1.35 | 0.20 | 0.61 |
|  | Median | 2.33 | 4.12 | 82.8 | 11.2 | 1.59 | 1.93 | 79.2 | 646 | - | 2.74 | 13.9 | 54.6 | 0.42 | - | - | 0.50 |
|  | Average | 2.29 | 4.11 | 68.9 | 14.4 | 1.94 | 1.93 | 74.6 | 784 | - | 3.07 | 12.2 | 45.3 | 0.43 | - | - | 0.50 |
|  | SD | 1.02 | 0.42 | 38.5 | 9.54 | 1.15 | 0.72 | 22.0 | 576 | - | 1.67 | 4.80 | 19.2 | 0.05 | - | - | 0.12 |
| Local grass - Jaffna (3) | Minimum | 0.82 | 2.01 | 8.48 | 8.40 | 0.68 | 0.42 | 83.5 | 256 | <LOD | 0.88 | 1.36 | 10.7 | 0.30 | <LOD | <LOD | 0.18 |
|  | Maximum | 56.8 | 7.44 | 67.3 | 38.6 | 1.70 | 1.87 | 180 | 466 | 1.07 | 2.41 | 6.02 | 27.0 | 0.54 | 2.17 | 0.22 | 0.38 |
|  | Median | 1.02 | 6.55 | 33.9 | 13.7 | 0.85 | 1.30 | 146 | 304 | - | 1.67 | 4.22 | 23.0 | 0.38 | - | - | 0.27 |
|  | Average | 19.5 | 5.66 | 32.6 | 17.2 | 1.12 | 1.32 | 135 | 357 | - | 1.66 | 4.30 | 20.4 | 0.40 | - | - | 0.28 |
|  | SD | 32.2 | 2.22 | 22.4 | 12.3 | 0.49 | 0.59 | 40.7 | 100 | - | 0.54 | 1.92 | 6.3 | 0.09 | - | - | 0.07 |
| Local grass - Mannar (3) | Minimum | 0.38 | 1.86 | 8.37 | 0.83 | <LOD | <LOD | 39.0 | 89.2 | <LOD | <LOD | 1.92 | 6.32 | <LOD | <LOD | <LOD | 0.07 |
|  | Maximum | 5.24 | 7.69 | 30.2 | 12.3 | 0.85 | 1.38 | 192 | 214 | 1.68 | 1.23 | 6.43 | 71.8 | 0.35 | 2.04 | 0.10 | 0.44 |
|  | Median | 3.01 | 2.36 | 24.1 | 2.58 | - | - | 59.4 | 100 | - | - | 3.25 | 18.6 | - | - | - | 0.37 |
|  | Average | 2.88 | 3.37 | 22.0 | 4.51 | - | - | 92.5 | 122 | - | - | 3.67 | 26.1 | - | - | - | 0.30 |
|  | SD | 2.43 | 2.43 | 8.14 | 4.54 | - | - | 67.2 | 52 | - | - | 1.70 | 26.1 | - | - | - | 0.16 |
| Local grass – Karunagala (14) | Minimum | 0.27 | 1.93 | 14.2 | 2.26 | <LOD | 0.92 | 54.8 | 49.8 | <LOD | 1.17 | 1.98 | 20.7 | <LOD | <LOD | <LOD | <LOD |
|  | Maximum | 2.61 | 4.99 | 34.7 | 31.9 | 6.18 | 10.28 | 883 | 947 | 2.31 | 3.78 | 37.6 | 56.7 | 0.65 | 1.67 | 0.70 | 0.58 |
|  | Median | 0.58 | 3.81 | 20.6 | 4.46 | - | 1.68 | 171 | 152 | - | 1.87 | 3.98 | 25.1 | - | - | - | - |
|  | Average | 0.81 | 3.36 | 23.4 | 6.48 | - | 2.34 | 258 | 284 | - | 2.00 | 6.91 | 28.7 | - | - | - | - |
|  | SD | 0.63 | 0.95 | 7.0 | 7.75 | - | 2.37 | 231 | 280 | - | 0.75 | 9.05 | 10.3 | - | - | - | - |

**Table S10-** Summary results for all analytes in the concentrates used by local dairy farmers in the sampling area,
(Vavuniya, Mannar, Jaffna and Kurunegala, in Sri Lanka).

| **Feed (number of samples)** | **Parameter** | **Elemental concentrations** | | | | | | | | | | | | | | | |
| --- | --- | --- | --- | --- | --- | --- | --- | --- | --- | --- | --- | --- | --- | --- | --- | --- | --- |
|  |  | **Major elements (g/kg d.w)** | | | | **Trace elements (mg/kg d.w)** | | | | | | | | | | | |
|  |  | **Na** | **Mg** | **K** | **Ca** | **V** | **Cr** | **Mn** | **Fe** | **Co** | **Ni** | **Cu** | **Zn** | **Se** | **Mo** | **Cd** | **Pb** |
| Beer waste (4) | Minimum | 0.25 | 1.27 | 0.63 | 2.03 | <LOD | 0.74 | 49.4 | 146 | <LOD | <LOD | 12.9 | 125 | 0.29 | 2.59 | <LOD | 0.19 |
|  | Maximum | 0.48 | 2.63 | 0.93 | 3.28 | <LOD | 110 | 74.3 | 353 | <LOD | 1.20 | 25.9 | 189 | 0.50 | 3.41 | 0.28 | 0.91 |
|  | Median | 0.32 | 2.09 | 0.69 | 3.02 | - | 19.4 | 68.5 | 225 | - | - | 18.0 | 128 | 0.37 | 3.02 | - | 0.30 |
|  | Average | 0.34 | 2.02 | 0.73 | 2.84 | - | 37.3 | 65.2 | 238 | - | - | 18.7 | 142 | 0.38 | 3.01 | - | 0.42 |
|  | SD | 0.11 | 0.56 | 0.14 | 0.57 | - | 50.7 | 10.8 | 86 | - | - | 6.7 | 31 | 0.09 | 0.35 | - | 0.33 |
| CIC cow feed (11) | Minimum | 0.12 | 2.76 | 8.70 | 0.45 | <LOD | <LOD | 94.0 | 132 | <LOD | 1.04 | 9.25 | 52.9 | <LOD | <LOD | <LOD | 0.25 |
|  | Maximum | 2.80 | 4.28 | 10.93 | 10.7 | <LOD | 1.05 | 163 | 224 | 0.86 | 3.00 | 23.1 | 120 | 0.67 | <LOD | <LOD | 4.46 |
|  | Median | 0.64 | 3.53 | 9.57 | 2.41 | - | - | 106 | 181 | - | 1.39 | 11.2 | 66.2 | - | - | - | 0.42 |
|  | Average | 1.32 | 3.46 | 9.63 | 4.78 | - | - | 114 | 181 | - | 1.66 | 13.3 | 74.0 | - | - | - | 0.76 |
|  | SD | 1.27 | 0.39 | 0.75 | 4.50 | - | - | 21 | 24 | - | 0.71 | 4.6 | 21.2 | - | - | - | 1.23 |
| Prima milk mash (2) | Minimum | 1.28 | 4.77 | 12.4 | 5.85 | <LOD | 0.73 | 130 | 257 | <LOD | 1.12 | 12.0 | 83.8 | 0.49 | <LOD | <LOD | 0.29 |
|  | Maximum | 2.58 | 4.80 | 13.1 | 9.69 | <LOD | 1.54 | 137 | 300 | 0.62 | 1.24 | 14.9 | 99.6 | 0.76 | <LOD | <LOD | 0.48 |
|  | Median | 1.93 | 4.78 | 12.8 | 7.77 | - | 1.14 | 134 | 279 | - | 1.18 | 13.4 | 91.7 | 0.62 | - | - | 0.38 |
|  | Average | 1.93 | 4.78 | 12.8 | 7.77 | - | 1.14 | 134 | 279 | - | 1.18 | 13.4 | 91.7 | 0.62 | - | - | 0.38 |
|  | SD | 0.92 | 0.02 | 0.5 | 2.72 | - | 0.57 | 4 | 30.5 | - | 0.09 | 2.1 | 11.1 | 0.19 | - | - | 0.13 |
| Rice bran (25) | Minimum | <LOD | 4.49 | 6.94 | <LOD | <LOD | <LOD | 83.1 | 54.3 | <LOD | <LOD | 3.21 | 27.7 | <LOD | <LOD | <LOD | <LOD |
|  | Maximum | 0.82 | 8.54 | 15.2 | 2.08 | 0.70 | <LOD | 206 | 327 | 0.97 | 6.08 | 27.6 | 101 | 0.57 | <LOD | 0.27 | 0.42 |
|  | Median | - | 6.21 | 12.1 | - | - | - | 115 | 90.9 | - | - | 6.15 | 46.8 | - | - | - | - |
|  | Average | - | 6.38 | 11.7 | - | - | - | 117 | 106 | - | - | 7.26 | 50.4 | - | - | - | - |
|  | SD | - | 1.17 | 2.1 | - | - | - | 27 | 64.2 | - | - | 4.71 | 15.6 | - | - | - | - |
| Rice polish (10) | Minimum | 0.14 | 2.67 | 4.76 | 0.19 | <LOD | <LOD | 103 | 102 | <LOD | <LOD | 2.75 | 36.0 | 0.31 | <LOD | <LOD | 0.19 |
|  | Maximum | 0.62 | 10.4 | 12.0 | 2.26 | 0.71 | <LOD | 427 | 280 | 1.05 | 1.41 | 43.8 | 134 | 0.69 | <LOD | <LOD | 0.40 |
|  | Median | 0.18 | 4.60 | 6.16 | 0.51 | - | - | 228 | 191 | - | - | 3.92 | 53.9 | 0.42 | - | - | 0.27 |
|  | Average | 0.24 | 5.17 | 6.94 | 0.62 | - | - | 230 | 189 | - | - | 8.50 | 63.7 | 0.44 | - | - | 0.28 |
|  | SD | 0.14 | 2.34 | 2.35 | 0.61 | - | - | 89 | 78.0 | - | - | 12.5 | 30.0 | 0.11 | - | - | 0.07 |
| Wheat bran (2) | Minimum | 0.14 | 4.37 | 9.77 | 0.73 | <LOD | <LOD | 102 | 152 | <LOD | 0.94 | 10.6 | 54.0 | 0.32 | <LOD | <LOD | 0.21 |
|  | Maximum | 3.31 | 5.34 | 14.7 | 8.89 | 1.91 | 1.19 | 125 | 490 | <LOD | 3.18 | 13. 6 | 81.3 | 0.42 | <LOD | <LOD | 0.52 |
|  | Median | 1.73 | 4.86 | 12.3 | 4.81 | - | - | 114 | 321 | - | 2.06 | 12.1 | 67.7 | 0.37 | - | - | 0.36 |
|  | Average | 1.73 | 4.86 | 12.3 | 4.81 | - | - | 114 | 321 | - | 2.06 | 12.1 | 67.7 | 0.37 | - | - | 0.36 |
|  | SD | 2.24 | 0.68 | 3.52 | 5.76 | - | - | 16 | 239 | - | 1.59 | 2.1 | 19.3 | 0.07 | - | - | 0.22 |

**Table S10-** Continuation.

| **Feed (number of samples)** | **Parameter** | **Elemental concentrations** | | | | | | | | | | | | | | | |
| --- | --- | --- | --- | --- | --- | --- | --- | --- | --- | --- | --- | --- | --- | --- | --- | --- | --- |
|  |  | **Major elements (g/kg d.w)** | | | | **Trace elements (mg/kg d.w)** | | | | | | | | | | | |
|  |  | **Na** | **Mg** | **K** | **Ca** | **V** | **Cr** | **Mn** | **Fe** | **Co** | **Ni** | **Cu** | **Zn** | **Se** | **Mo** | **Cd** | **Pb** |
| Homemade mix -Vavuniya (4) | Minimum | 0.10 | 5.85 | 5.89 | 0.06 | <LOD | <LOD | 111 | 68.0 | <LOD | <LOD | 5.87 | 40.0 | 0.38 | <LOD | <LOD | <LOD |
|  | Maximum | 1.73 | 7.68 | 13.5 | 7.38 | 2.15 | 1.14 | 298 | 552 | 0.90 | 3.24 | 21.7 | 186 | 1.11 | <LOD | <LOD | 0.78 |
|  | Median | 0.46 | 7.20 | 11.8 | 2.02 | - | - | 154 | 75.2 | - | - | 11.6 | 75.7 | 0.54 | - | - | - |
|  | Average | 0.69 | 6.98 | 10.8 | 2.87 | - | - | 179 | 193 | - | - | 12.7 | 94.3 | 0.64 | - | - | - |
|  | SD | 0.73 | 0.82 | 3.6 | 3.20 | - | - | 82 | 240 | - | - | 7.8 | 65.8 | 0.32 | - | - | - |
| Homemade mix - Jaffna (3) | Minimum | 0.23 | 4.46 | 8.48 | 2.47 | <LOD | <LOD | 45. 8 | 87.4 | <LOD | <LOD | 5.68 | 28.4 | 0.31 | <LOD | <LOD | 0.21 |
|  | Maximum | 0.66 | 8.67 | 13. 7 | 13.7 | <LOD | <LOD | 94.7 | 178 | <LOD | 1.95 | 19.9 | 90.8 | 0.72 | <LOD | <LOD | 0.39 |
|  | Median | 0.65 | 4.90 | 9.07 | 2.49 | - | - | 56.8 | 95.5 | - | - | 6.0 | 37.4 | 0.41 | - | - | 0.24 |
|  | Average | 0.51 | 6.01 | 10.4 | 6.23 | - | - | 65.7 | 120 | - | - | 10.5 | 52.2 | 0.48 | - | - | 0.28 |
|  | SD | 0.24 | 2.31 | 2.8 | 6.50 | - | - | 25.7 | 50 | - | - | 8.1 | 33.7 | 0.21 | - | - | 0.09 |

**Table S11-** Summary results for all analytes in the nutritional supplements used by local dairy farmers in the sampling area,
(Vavuniya, Mannar, Jaffna and Kurunegala, in Sri Lanka).

| **Region (number of samples)** | **Parameter** | **Elemental concentrations** | | | | | | | | | | | | | | | |
| --- | --- | --- | --- | --- | --- | --- | --- | --- | --- | --- | --- | --- | --- | --- | --- | --- | --- |
|  |  | **Major elements (g/kg d.w)** | | | | **Trace elements (mg/kg d.w)** | | | | | | | | | | | |
|  |  | **Na** | **Mg** | **K** | **Ca** | **V** | **Cr** | **Mn** | **Fe** | **Co** | **Ni** | **Cu** | **Zn** | **Se** | **Mo** | **Cd** | **Pb** |
| Aminol (2) | Minimum | 106 | 16.0 | 12.0 | 154 | 5.07 | 3.49 | 255 | 220 | 15.2 | 5.85 | 819 | 7180 | 28.9 | <LOD | 0.22 | 1.46 |
|  | Maximum | 112 | 17.2 | 12. 8 | 157 | 15.9 | 17.0 | 378 | 3658 | 21. 8 | 6.48 | 1276 | 7461 | 29.8 | <LOD | 0.38 | 5.79 |
|  | Median | 109 | 16.5 | 12.4 | 155 | 10.5 | 10.2 | 316 | 1939 | 18.5 | 6.16 | 1048 | 7320 | 29.4 | - | 0.30 | 3.62 |
|  | Average | 109 | 16.6 | 12.4 | 155 | 10.5 | 10.2 | 316 | 1939 | 18.5 | 6.16 | 1048 | 7320 | 29.4 | - | 0.30 | 3.62 |
|  | SD | 4 | 0.8 | 0.6 | 2 | 7.6 | 9.6 | 87 | 2431 | 4.7 | 0.44 | 323 | 199 | 0.7 | - | 0.12 | 3.06 |
| Brander Primer (2) | Minimum | 0.21 | 4.16 | 11.9 | 1.26 | <LOD | 0.90 | 114 | 212 | <LOD | 1.91 | 12.1 | 69.7 | 0.32 | <LOD | <LOD | 0.25 |
|  | Maximum | 2.09 | 4.90 | 12.7 | 10.4 | 0.71 | 1.03 | 143 | 497 | 2.97 | 2.07 | 27.2 | 107 | 0.61 | <LOD | <LOD | 0.34 |
|  | Median | 1.15 | 4.53 | 12.3 | 5.84 | - | 0.96 | 128 | 354 | - | 1.99 | 19.6 | 88.5 | 0.47 | - | - | 0.30 |
|  | Average | 1.15 | 4.53 | 12.3 | 5.84 | - | 0.96 | 128 | 354 | - | 1.99 | 19.6 | 88.5 | 0.47 | - | - | 0.30 |
|  | SD | 1.33 | 0.52 | 0.5 | 6.48 | - | 0.10 | 20 | 201 | - | 0.11 | 10.6 | 26.5 | 0.20 | - | - | 0.06 |
| Calcium powder/ limestone (9) | Minimum | 2.65 | 6.31 | 0.43 | 136 | 15.16 | 5.46 | 526 | 447 | 11.6 | 14.0 | 1029 | 5303 | 3.90 | <LOD | 0.38 | 2.80 |
|  | Maximum | 54.8 | 29.3 | 1.32 | 263 | 74.2 | 48.0 | 6685 | 2723 | 207 | 27.3 | 3975 | 9032 | 108 | 3.18 | 7.59 | 39.16 |
|  | Median | 28.0 | 17.2 | 0.56 | 172 | 35.6 | 7.81 | 3458 | 1217 | 34.9 | 17.7 | 2479 | 6790 | 35.7 | - | 0.68 | 4.35 |
|  | Average | 26.7 | 17.0 | 0.71 | 187 | 37.9 | 16.2 | 3916 | 1408 | 61.4 | 19.4 | 2426 | 6612 | 41.6 | - | 2.09 | 12.66 |
|  | SD | 21.8 | 9.6 | 0.29 | 40 | 22.3 | 16.6 | 2339 | 834 | 62.8 | 4.9 | 1295 | 1154 | 34.0 | - | 2.84 | 14.19 |
| Supermineral mix (2) | Minimum | 1.28 | 4.77 | 12.4 | 5.85 | <LOD | 0.73 | 130 | 257 | <LOD | 1.12 | 12.0 | 83.8 | 0.49 | <LOD | <LOD | 0.29 |
|  | Maximum | 2.58 | 4.80 | 13.1 | 9.69 | <LOD | 1.54 | 137 | 300 | 0.62 | 1.24 | 14.9 | 99.6 | 0.76 | <LOD | <LOD | 0.48 |
|  | Median | 1.93 | 4.78 | 12.8 | 7.77 | - | 1.14 | 134 | 279 | - | 1.18 | 13.4 | 91.7 | 0.62 | - | - | 0.38 |
|  | Average | 1.93 | 4.78 | 12.8 | 7.77 | - | 1.14 | 134 | 279 | - | 1.18 | 13.4 | 91.67 | 0.62 | - | - | 0.38 |
|  | SD | 0.92 | 0.02 | 0.48 | 2.72 | - | 0.57 | 4 | 30 | - | 0.09 | 2.1 | 11.1 | 0.19 | - | - | 0.13 |
